# Coupling Fast Zn^2+^ Kinetics With V–O Framework Stabilization via Nitrogen‐Rich Interfacial Engineering of Spinel ZnV_2_O_4_

**DOI:** 10.1002/advs.78612

**Published:** 2026-11-09

**Authors:** Ashok Kumar Kakarla, Incheol Jeong, Chanho Lee, SeungEun Yu, Ryeowon Kang, Binna Kim, Wooseop Jo, San Moon, Patrick Joohyun Kim, Seho Sun, Chanho Kim, Ki‐Min Roh, Junghyun Choi, Dongsoo Lee

**Affiliations:** ^1^ School of Chemical Biological & Battery Engineering Gachon University Gyeonggi‐do Republic of Korea; ^2^ Resources Utilization Research Center, KIGAM Daejeon Republic of Korea; ^3^ Advanced Battery Research Center Korea Research Institute of Chemical Technology Daejeon Republic of Korea; ^4^ Department of Chemical and Biological Engineering Sookmyung Women's University Seoul Republic of Korea; ^5^ School of Chemical Engineering Yeungnam University Gyeongsan Republic of Korea; ^6^ Chemical Science Division, Oak Ridge National Laboratory Oak Ridge Tennessee USA

**Keywords:** aqueous zinc‐ion batteries, interfacial charge transfer, nitrogen‐rich interfacial regulator, spinel ZnV_2_O_4_, surface‐dominated charge‐storage kinetics, V─O framework stabilization

## Abstract

Aqueous zinc‐ion batteries (AZIBs) offer intrinsic safety and cost advantages, yet spinel zinc vanadate cathodes remain limited by sluggish interfacial Zn^2+^ storage kinetics, polarization, and structural degradation. Herein, graphitic carbon nitride (GCN) is employed as a nitrogen‐rich interfacial regulator (NIR) to construct NIR‐integrated spinel ZnV_2_O_4_ (ZVO) cathodes (GZVO) through ultrasonication‐assisted physical integration. The GCN‐derived NIR provides polar N sites and regulated interfacial association with ZVO, promoting interfacial Zn^2+^ adsorption/migration and charge transfer while mitigating local framework distortion. The optimized GZVO‐7 wt.% cathode delivers 405 mAh g^−1^ after 150 cycles at 0.5 A g^−1^, retains 258.93 mAh g^−1^ at 10 A g^−1^, and maintains 218.24 mAh g^−1^ after 3000 cycles at 5 A g^−1^. Cyclic voltammetry (CV), in situ electrochemical impedance spectroscopy–distribution of relaxation times (EIS‐DRT), and galvanostatic intermittent titration technique (GITT) reveal enhanced pseudocapacitive contribution, reduced polarization, and reversible Zn^2+^ storage. Ex situ x‐ray diffraction (XRD) and x‐ray photoelectron spectroscopy (XPS) reveal persistence and partial recovery of ZVO‐derived crystalline domains together with surface V^5+^/V^4+^ redox evolution, oxygen‐environment modulation, and N‐site‐mediated Zn^2+^ interfacial regulation. Density functional theory (DFT) verifies favorable surface Zn‐removal energetics and reinforced V─O bonding. This work establishes NIR as an effective strategy for durable, high‐rate vanadate cathodes in AZIBs.

## Graphical abstract

Graphitic carbon nitride acts as a nitrogen‐rich interfacial regulator for spinel ZnV_2_O_4_ cathodes in aqueous zinc‐ion batteries. Polar N sites promote interfacial Zn^2+^ adsorption, migration, and charge transfer, while mitigating local framework distortion and reinforcing V─O bonding. This dual role couples fast surface‐dominated Zn^2+^ storage kinetics with reversible V^5+^/V^4+^ redox, enabling durable, high‐rate vanadate cathodes.

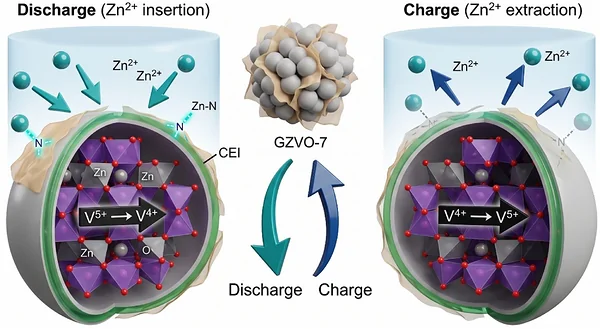

## 1 Introduction

Aqueous zinc–ion batteries (AZIBs) are promising candidates for next‐generation large‐scale energy storage owing to the high theoretical capacity of metallic Zn, low cost, intrinsic safety, and environmental compatibility of mildly acidic aqueous electrolytes [1, 2, 3, 4, 5]. Compared with conventional nonaqueous battery systems, AZIBs offer reduced flammability risks and improved resource sustainability, which have stimulated extensive efforts in electrode and electrolyte engineering [6, 7]. However, the practical development of AZIBs remains largely limited by cathode instability. Typical cathode families, including manganese oxides, vanadium‐based compounds, Prussian blue analogues, and organic/conversion‐type hosts, commonly suffer from poor intrinsic conductivity, sluggish Zn^2+^ diffusion, dissolution of active species, structural degradation, and complicated Zn^2+^/H^+^ co‐insertion chemistry, resulting in rapid capacity decay [8]. Recent studies further reveal that electrolyte‐induced phase transformations and activation processes can initially increase capacity but often accelerate side reactions and structural instability during prolonged cycling [9, 10, 11, 12]. Therefore, constructing cathodes with robust frameworks, rapid Zn^2+^ transport, and regulated interfacial chemistry remains essential for durable and high‐rate AZIBs.

Among various cathode candidates, vanadium‐based materials are particularly attractive because of their multivalent V^5+^/V^4+^/V^3+^ redox chemistry, diverse crystal structures, and abundant ion‐storage sites [13, 14]. Layered vanadium oxides, vanadium bronzes, spinel‐type zinc vanadates, polyanion‐type vanadium phosphates, and vanadium‐based Prussian blue analogues have demonstrated high capacities and broad electrochemical activity in aqueous Zn systems [9, 15, 16, 17, 18, 19, 20]. Nevertheless, many vanadium‐based cathodes still experience insufficient electronic conductivity, slow Zn^2+^ migration, vanadium dissolution, and uncontrolled structural evolution in aqueous electrolytes. For example, some Zn‐containing vanadates and vanadium hexacyanoferrate cathodes undergo electrolyte‐driven reconstruction or dissolution–redeposition processes during cycling, which complicate the charge‐storage mechanism and compromise long‐term stability [9, 12, 17]. Although heteroatom doping, defect regulation, and polyanion framework design have been explored to improve Zn^2+^ diffusion and structural reversibility [1, 14, 21, 22, 23, 24], achieving simultaneous kinetic acceleration and framework stabilization remains challenging.

Carbon integration and graphitic carbon nitride (g‐C_3_N_4_) modification have previously been used to improve oxide cathodes for AZIBs, although their preparation routes and functional mechanisms differ. MOF‐derived porous ZnV_2_O_4_@C is produced through pyrolysis of a Zn/V‐containing precursor, generating an integrated conductive‐carbon phase that improves electronic transport, exposes additional reaction sites, and stabilizes the ion‐storage channels [25]. Hierarchical ZnV_2_O_4_@C with a carbon shell has similarly been reported to suppress vanadium dissolution and shorten ion‐diffusion distances; importantly, this material undergoes electrochemically induced phase reconstruction during the first cycle [26]. N‐doped VO_x_@g‐C_3_N_4_ is instead prepared through in situ growth, with the reported incorporation of N into VO_2_ nanorods contributing to conductivity and ion‐diffusion enhancement [27]. In manganese‐based systems, g‐C_3_N_4_‐coated α‐MnO_2_ hollow nanorods use a coating/heterojunction configuration to accommodate structural changes and improve cycling stability [28], whereas g‐C_3_N_4_‐modified spinel ZnMn_2_O_4_ is formed through a one‐step solvothermal process and is reported to increase active‐site exposure, charge transfer, and structural stability [29]. In contrast to these in situ‐grown, pyrolysis‐derived, or coated architectures, the present nitrogen rich interfacial regulator (NIR) integrated spinel ZnV_2_O_4_ (GZVO) composites are prepared by ex situ aqueous mixing of separately synthesized ZVO and g‐C_3_N_4_ (GCN) through ultrasonication, stirring, and drying. Therefore, GZVO is described as a physically integrated composite with interfacial association rather than as a chemically defined heterojunction or a continuously coated structure. The term NIR is used as a functional descriptor for the proposed role of the nitrogen‐rich GCN component at the ZVO (ZnV_2_O_4_)/electrolyte interface and does not, by itself, establish direct chemical bonding. The present contribution is distinguished by the systematic control of the GCN fraction at 5, 7, and 10 wt.% and by correlating the optimized 7 wt.% composition with electrochemical kinetics, interfacial polarization, structural evolution, and theoretical analysis.

Herein, spinel ZVO nanoparticles are integrated with a GCN‐derived NIR to construct GZVO cathodes with different NIR contents of 5, 7, and 10 wt.%. ZVO is synthesized through a solvothermal route followed by Ar‐assisted calcination, and the GZVO composites are obtained through mild ultrasonication‐assisted physical integration. Based on the synthesis route and structural evidence, the GZVO architecture is described as ZVO nanoparticles interfacially associated with the NIR backbone, rather than as a continuous GCN coating layer. Among the prepared electrodes, GZVO‐7 exhibits the optimal balance between active ZVO exposure, interfacial contact, charge‐transfer pathways, and Zn^2+^ accessibility, leading to enhanced reversible capacity, rate capability, and long‐term cycling stability. DFT calculations are employed to clarify the role of the NIR interface in lowering the relative surface Zn‐removal energy and reinforcing the V–O framework, while cyclic voltammetry (CV), in situ electrochemical impedance spectroscopy–distribution of relaxation times analysis (EIS‐DRT), and galvanostatic intermittent titration technique (GITT) are used to probe reaction kinetics, interfacial polarization, and Zn^2+^ diffusion behavior. Furthermore, ex situ x‐ray diffraction (XRD), x‐ray photoelectron spectroscopy (XPS), and post‐cycling structural analyses reveal the reversible Zn^2+^ storage mechanism, vanadium redox evolution, oxygen‐environment modulation, and interfacial stability of the optimized cathode. This work highlights NIR as a viable strategy for simultaneously improving Zn^2+^ transport kinetics and structural durability in spinel vanadate cathodes for AZIBs.

## 2 Results and Discussion

### 2.1 Structural Characterization

Figure 1 summarizes the synthesis strategy and interfacial design concept of the nitrogen‐rich interfacial regulator (NIR)‐integrated spinel ZnV_2_O_4_ (GZVO) cathode. As shown in Figure 1a, spinel ZnV_2_O_4_ (ZVO) was prepared through a solvothermal reaction followed by Ar‐assisted thermal conversion, while graphitic carbon nitride (g‐C_3_N_4_, GCN) was obtained by urea‐derived thermal polymerization under N_2_. Because ZVO and GCN were synthesized separately and subsequently combined through ultrasonication, stirring, and drying, GZVO is defined here as a physically integrated composite in which ZVO nanoparticles are interfacially associated with the GCN framework. In this manuscript, NIR denotes the proposed functional role of nitrogen‐rich GCN at the ZVO/electrolyte interface; the term does not, by itself, imply in situ growth, a continuous coating, covalent ZVO–GCN bonding, or the formation of a chemically defined heterojunction. Subsequent ultrasonication‐assisted physical integration enabled the interfacial association of ZVO nanoparticles with the GCN‐derived NIR framework, yielding GZVO composites with regulated interfacial architectures. In this design, the NIR is introduced as a chemically active interfacial component rather than a conventional conductive‐carbon additive [30, 31, 32]. The proposed GZVO interfacial configuration is illustrated in Figure 1b. The polar N sites within the NIR framework are expected to regulate the local ZVO/electrolyte interface and provide favorable environments for Zn^2+^ interaction. As schematically compared in Figure 1c, d, pristine ZVO is prone to sluggish Zn^2+^ transport and interfacial degradation, whereas the NIR‐integrated GZVO architecture is designed to improve interfacial accessibility and structural durability during repeated Zn^2+^ insertion/extraction. The detailed chemical, kinetic, and theoretical evidence for this design is discussed in the following sections.

**FIGURE 1:**
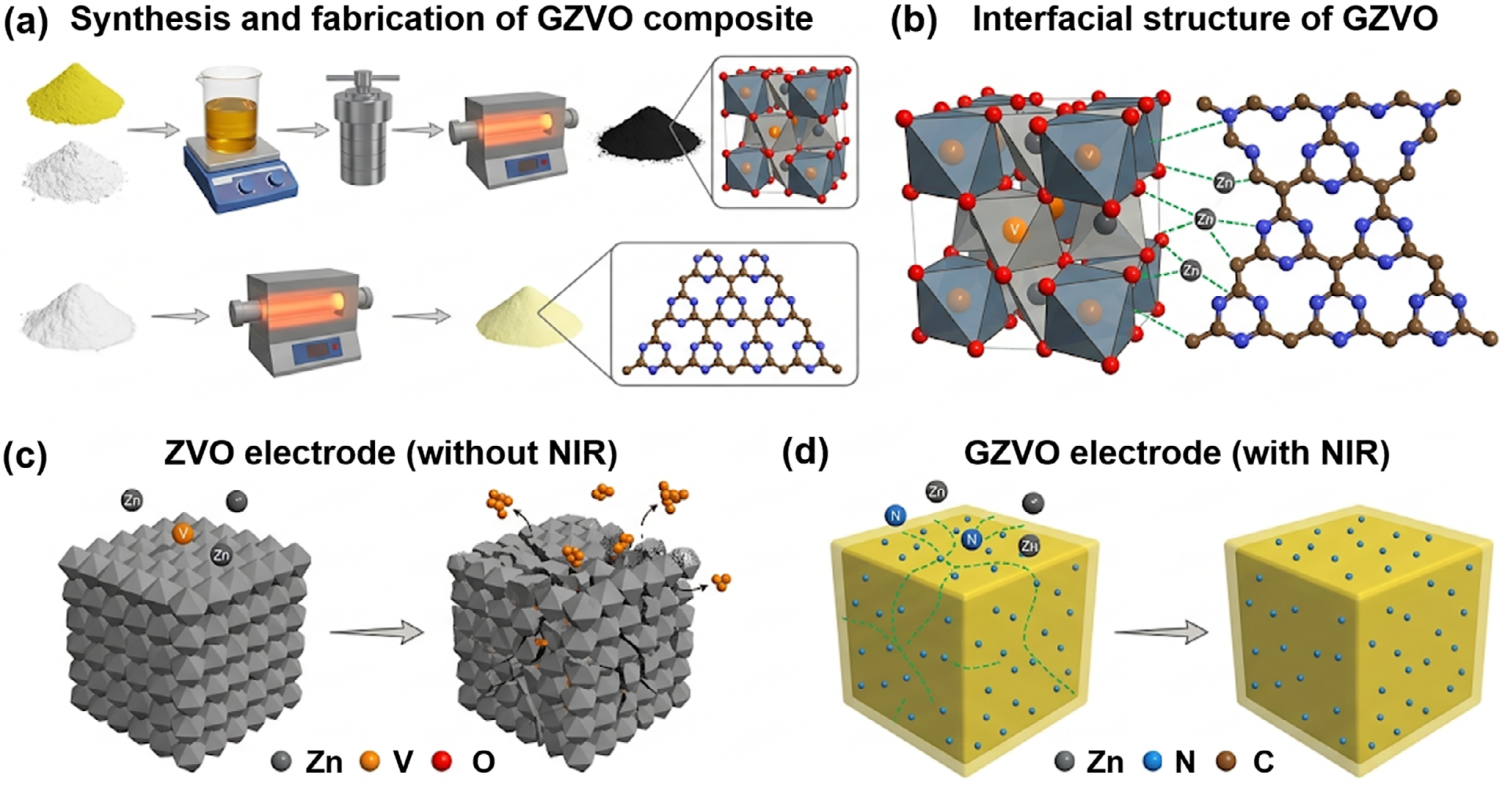
Schematic illustration of the synthesis, interfacial design, and proposed stabilization mechanism of the NIR‐integrated GZVO cathode. (a) Synthetic route for spinel ZVO, graphitic carbon nitride (GCN), and GZVO composites prepared by ultrasonication‐assisted physical integration. (b) Proposed interfacial configuration between ZVO and the nitrogen‐rich GCN framework, highlighting the role of polar N sites in regulating local Zn^2+^ transport and interfacial electronic structure. (c, d) Schematic comparison of pristine ZVO and GZVO electrodes during cycling.

The crystallographic structures of ZVO and the GZVO‐x (x = 5, 7, 10 wt.%) composites were examined by PXRD, as shown in Figure 2a. The major diffraction reflections of pristine ZVO can be indexed to cubic spinel ZnV_2_O_4_ (PDF# 75–0318, space group Fd‐3m), and these dominant reflections are retained after integration with the GCN‐derived NIR [33]. The sharp and well‐defined reflections confirm the high crystallinity of ZVO after calcination at 800°C, indicating the successful formation of the spinel ZVO phase through the solvothermal–calcination route. Because the precursor mixture contained a higher Zn content than required for stoichiometric ZVO, the elemental Zn/V ratio of the isolated ZVO material was evaluated by Inductively Coupled Plasma Optical Emission Spectroscopy (ICP‐OES). The measured V and Zn concentrations in the same analysed solution were 1.7765 and 2.8722 mg kg^−1^, respectively. Conversion of these mass concentrations to molar quantities produced a Zn:V ratio of approximately 1.26:1 (Figure S1). The corresponding Zn molar ratios of GZVO‐5 wt.%, GZVO‐7 wt.%, and GZVO‐10 wt.% were approximately 1.25:1, 1.53:1, and 1.27:1, respectively, as summarized in Table S1. All four measured ratios are higher than the ideal Zn:V ratio of 0.50:1 for stoichiometric ZVO, consistently demonstrating the Zn‐rich composition of the synthesized materials. The variation among the composite ratios is non‐monotonic with GCN loading and is therefore not interpreted as a systematic GCN‐induced modification of the Zn/V stoichiometry, particularly because GCN introduces neither Zn nor V during the physical integration process. These results demonstrate that the experimental ZVO material is Zn‐rich and that the excess Zn introduced during synthesis is not completely removed during product recovery. Nevertheless, the PXRD pattern is dominated by reflections indexed to cubic spinel ZVO, and no distinct reflections attributable to crystalline ZnO or another Zn‐rich secondary phase are detected. Therefore, the retained excess Zn may be associated with x‐ray‐amorphous, nanocrystalline, surface‐associated, or defect‐related Zn‐containing environments that cannot be distinguished unambiguously using the present measurements. Accordingly, the experimental material is described as a Zn‐rich ZVO material with a ZVO ‐dominant crystalline phase rather than as compositionally pure stoichiometric ZVO In the ideal cubic spinel ZVO structure, Zn^2+^ occupies the tetrahedral A sites, whereas V^3+^ occupies the octahedral B sites within a cubic close‐packed oxygen framework, forming a 3D lattice favourable for reversible Zn^2+^ storage [34]. After integration with NIR, the diffraction patterns of GZVO‐x (x = 5, 7, 10 wt.%) remain highly consistent with those of pristine ZVO, without discernible peak shifts or additional crystalline impurity phases. This indicates that the dominant spinel ZVO ‐derived crystalline framework is retained after ultrasonication‐assisted physical integration. A weak reflection near 37° is discernible in the GZVO‐x (x = 5, 7, 10 wt.%) patterns. Comparison with the ZnV_2_O_4_ reference pattern (PDF# 75–0318) shows that this feature coincides with the low‐intensity (222) reflection of cubic spinel ZVO and is therefore not assigned to GCN or an additional crystalline impurity phase. The apparently greater visibility of this weak reflection in the vertically offset composite patterns is associated with differences in relative peak intensity and graphical presentation. In contrast, the characteristic diffraction reflections of GCN, generally observed near 13.1° for the (100) plane and 27.4° for the (002) plane, are not independently resolved in the GZVO composites because of the relatively low NIR content and the weak and broad diffraction contribution of GCN [35]. The GCN fractions were controlled gravimetrically during composite preparation. Because the entire GCN–ZVO suspension was dried without filtration, centrifugation, or washing after mixing, the weighed GCN fractions define nominal loadings of 5, 7, and 10 wt.%. Based on the theoretical nitrogen mass fraction of ideal GCN, namely 60.86 wt.%, these loadings correspond to calculated nominal nitrogen contents of 3.04, 4.26, and 6.09 wt.% for GZVO‐5 wt.%, GZVO‐7 wt.%, and GZVO‐10 wt.%, respectively. No other unindexed crystalline reflections or systematic shifts in the principal ZnV_2_O_4_ peaks are detected. Therefore, within the detection limit of PXRD, the results indicate that NIR integration does not generate a detectable secondary crystalline phase or induce bulk crystallographic reconstruction of ZVO. Accordingly, the electrochemical differences among the ZVO and GZVO‐x (x = 5, 7, 10 wt.%) composite electrodes are not attributable to changes in the dominant crystalline phase but are more reasonably associated with NIR‐mediated interfacial regulation and transport modification.

**FIGURE 2:**
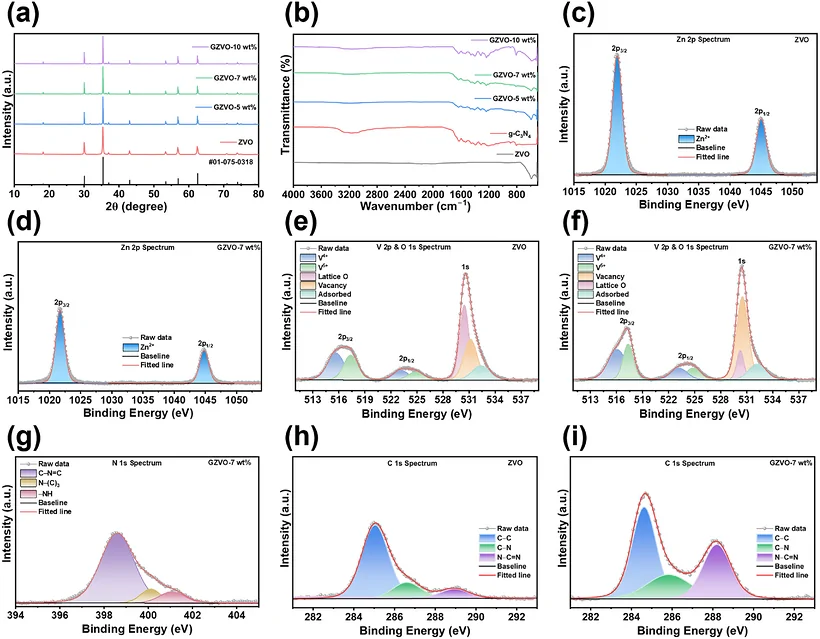
Structural and surface chemical characterization of ZVO and GZVO composites. (a) XRD patterns of ZVO, GZVO‐5 wt.%, GZVO‐7 wt.%, and GZVO‐10 wt.%. (b) FTIR spectra of ZVO, GCN, and GZVO composites. High‐resolution XPS spectra of (c, d) Zn 2p, (e, f) V 2p and O 1s, (g) N 1s, and (h, k) C 1s for ZVO and GZVO‐7 wt.%, respectively.

The surface functional groups and bonding environments of the synthesized samples were further examined by Fourier‐transform infrared (FT‐IR) spectroscopy over the range of 4000–500 cm^−1^, as shown in Figure 2b. The FT‐IR spectrum of pristine ZVO displays characteristic absorption bands at 509, 517, and 596 cm^−1^, which can be assigned to metal–oxygen vibrations associated with the spinel ZVO framework, further supporting the formation of the ZVO phase [36]. In comparison, the NIR exhibits typical vibrational features of graphitic carbon nitride, including bands at approximately 808 and 890 cm^−1^ corresponding to the breathing and out‐of‐plane bending modes of tri‐s‐triazine units, respectively. The absorption peaks located at 1236, 1310, 1389, 1452, 1536, and 1626 cm^−1^ are attributed to C─N and C═N stretching vibrations within the aromatic carbon nitride network [37]. In addition, the broad absorption band centered near 3163 cm^−1^ is associated with N─H/O─H stretching vibrations originating from terminal amino groups and adsorbed water. For the GZVO‐x (x = 5, 7, 10 wt.%) composites, the characteristic vibrational signals of both ZVO and NIR framework are retained, and the intensity of the carbon nitride‐related bands gradually increases with NIR content. These results support the successful incorporation of NIR into the GZVO composites while preserving the intrinsic metal–oxygen framework of ZVO and the molecular structure of the nitrogen‐rich carbon nitride component. The absence of newly emerged bands or pronounced peak shifts suggests that the GZVO composites are predominantly formed through physical interfacial integration rather than the formation of new bulk chemical phases. Together with the PXRD results, the FT‐IR analysis confirms the structural compatibility between ZVO and the NIR framework, providing chemical evidence for the coexistence of spinel ZVO and nitrogen‐rich carbon nitride motifs in the composite architecture.

XPS analysis was employed to elucidate the surface elemental composition and chemical‐state evolution of ZVO after integration with the NIR. Representative GZVO‐x (x = 5, 7, and 10 wt.%) composites were analyzed using pristine ZVO as a reference, and the fitted binding energies are summarized in Table 1. The survey spectra confirm the coexistence of Zn, V, O, C, and N in the GZVO composites, verifying the successful incorporation of the N‐containing framework into the ZVO‐based composite architecture (Figure S2). The high‐resolution Zn 2p spectra of all samples exhibit two characteristic peaks at 1021.6–1022.0 and 1044.7–1045.1 eV, corresponding to Zn 2p_3/2_ and Zn 2p_1/2_, respectively (Figure 2c, d and Figure S3). These binding energies are consistent with Zn^2+^ species in the ZVO framework. The absence of pronounced Zn 2p peak displacement after NIR integration indicates that the surface Zn chemical state is largely retained. The V 2p spectra can be deconvoluted into two pairs of spin–orbit doublets assigned to V^4+^ and V^5+^ species (Figure 2e,f and Figure S4). The peaks located at 515.6–516.3 and 523.2–523.8 eV are attributed to V^4+^ 2p_3/2_ and 2p_1/2_, whereas those centered at 517.2–517.6 and 524.8–525.2 eV correspond to V^5+^ 2p_3/2_ and 2p_1/2_, respectively [4, 13]. Although spinel ZVO contains nominal V^3+^ in the bulk framework, XPS mainly probes the near‐surface region. Therefore, the absence of an obvious V^3+^ component is attributed to surface oxidation of vanadium species during thermal treatment. The V^4+^/V^5+^ signals are thus interpreted as surface/interfacial vanadium states, while the PXRD results confirm the long‐range spinel ZVO structure. Compared with pristine ZVO, the GZVO composites show a modified surface V^4+^/V^5+^ distribution, suggesting that the NIR framework influences the surface electronic environment of ZVO. Among the investigated samples, GZVO‐7 wt.% exhibits a favorable mixed‐valence surface state, which may provide a more redox‐active interfacial environment. The O 1s spectra were fitted into three components centered at approximately 530.0, 531.1, and 532.5 eV (Figures 2e, f and Figure S4), corresponding to lattice oxygen in metal–oxygen bonds, defect‐related oxygen environments, and surface‐adsorbed oxygen‐containing species, respectively [13]. Compared with pristine ZVO, the GZVO composites show an increased defect‐related O 1s contribution, particularly for GZVO‐7 wt.%. This result suggests that NIR integration modulates the surface oxygen environment of ZVO and introduces more electrochemically accessible defect‐related surface sites. Such oxygen‐related surface environments can promote interfacial charge redistribution and facilitate Zn^2+^ storage kinetics.

**TABLE 1:** XPS binding energies and chemical‐state assignments of ZVO, GZVO‐5 wt.%, GZVO‐7 wt.%, and GZVO‐10 wt.% for Zn 2p, V 2p, O 1s, N 1s, and C 1s spectra.

Elements	ZVO	GZVO‐5 wt.%	GZVO‐7 wt.%	GZVO‐10 wt.%
**Zn**	1021.93, 1045.03	1021.64, 1044.75	1021.69, 1044.79	1022.03, 1045.12
**V**	515.65 (V^4+^), 517.35 (V^5+^), 523.16 (V^4+^), 524.83 (V^5+^)	515.83 (V^4+^), 517.42 (V^5+^), 523.27 (V^4+^), 524.75 (V^5+^)	516.06 (V^4+^), 517.54 (V^5+^), 523.21 (V^4+^), 524.81 (V^5+^)	516.17 (V^4+^), 517.32 (V^5+^), 523.18 (V^4+^), 525.19 (V^5+^)
**O**	530.44 (lattice O), 531.15 (Vacancy), 532.33 (Adsorbed)	530.26 (lattice O), 530.62 (Vacancy), 532.18 (Adsorbed)	530.26 (lattice O), 530.50 (Vacancy), 532.18 (Adsorbed)	530.46 (lattice O), 530.19 (Vacancy), 533.49 (Adsorbed)
**N**	NA	398.50 (C─N═C), 399.82 (N─(C)_3_), 400.69 (‐NH)	398.59 (C─N═C), 400.10 (N─(C)_3_), 401.07 (─NH)	398.72 (C─N═C), 399.91 (N─(C)_3_), 401.21 (─NH)
**C**	285.05 (C─C), 286.60 (C─O), 288.94 (O─C═O)	284.73 (C─C), 286.40 (C─N), 288.28 (N─C═N)	284.62 (C─C), 285.83 (C─N), 288.19 (N─C═N)	285.22 (C─C), 286.36 (C─N), 288.32 (N─C═N)

Further evidence for NIR incorporation is provided by the N 1s and C 1s spectra. As expected, pristine ZVO shows no detectable N 1s signal, whereas all GZVO composites display three characteristic N 1s components centered at approximately 398.5–398.7, 399.8–400.1, and 400.7–401.3 eV (Figure S5 and Figure 2g). These components are assigned to sp^2^‐hybridized C─N═C nitrogen, tertiary N─(C)_3_ nitrogen, and amino/protonated nitrogen species, respectively. These features are characteristic of the heptazine‐based carbon nitride framework. The qualitative increase in the N 1s signal across the composites is consistent with the increasing nominal NIR loading; however, because XPS is surface‐sensitive, it is not used to quantify the bulk nitrogen content or to demonstrate linear bulk‐composition scaling. To establish a reliable baseline for interpreting the nitrogen chemical environment, the high‐resolution N 1s spectrum of pristine GCN (Figure S6) was additionally measured and compared with that of GZVO‐7 wt.% (Figure 2g). Pristine GCN exhibits four components at 399.29, 400.98, 401.66, and 404.94 eV, corresponding to sp^2^‐hybridized C─N═C nitrogen, tertiary N─(C)_3_, terminal amino/protonated nitrogen, and a *π*‐excitation/charging‐related satellite feature, respectively. In GZVO‐7 wt.%, the corresponding framework‐N components are located at 398.59, 400.10, and 401.07 eV. The apparent shifts toward lower binding energy indicate that integration with ZVO modifies the local electronic environment of the GCN nitrogen species through interfacial charge redistribution. Importantly, because the component near 400–401 eV is already present in pristine GCN, it is assigned consistently to intrinsic tertiary N–(C)_3_ and is not considered independent evidence for the formation of a new covalent Zn–N phase. Instead, the collective N 1s shifts, together with the computational charge analysis discussed below, support electronic interaction between the polar N‐rich interface and the ZVO surface. In the C 1s spectra, pristine ZVO mainly exhibits a C─C/C═C component at 284.8 eV, originating from adventitious carbon and surface carbonaceous species (Figure 2h). In contrast, the GZVO composites exhibit an additional component at approximately 288.2–288.8 eV, corresponding to sp^2^‐bonded N─C═N species in the heptazine units of carbon nitride (Figure 2i and Figure S7). The simultaneous appearance of characteristic N 1s signals and the N─C═N component in C 1s confirms the successful integration of the NIR with ZVO. Therefore, the XPS results demonstrate that NIR integration preserves the Zn chemical state while modulating the surface vanadium valence distribution, oxygen‐related defect environment, and N‐containing interfacial chemistry of ZVO. In particular, GZVO‐7 wt.% exhibits an optimized combination of mixed‐valence vanadium species, enhanced defect‐related oxygen contribution, and abundant polar N‐containing groups, indicating a regulated surface electronic structure. These surface chemical features corroborate the NIR‐guided design concept proposed in Figure 1 and provide experimental support for improved interfacial charge redistribution, Zn^2+^‐affinity surface chemistry, and accelerated electrochemical kinetics in the optimized GZVO cathode.

The morphological and microstructural features of ZVO and GZVO‐x (x = 5, 7, and 10 wt.%) were investigated by scanning electron microscopy (SEM) and transmission electron microscopy (TEM), as shown in Figure 3. Figure 3a schematically illustrates the NIR‐content‐dependent interfacial decoration of pre‐formed ZVO aggregates. Because ZVO was calcined before ultrasonication‐assisted integration with GCN, the primary ZVO aggregate structure is mainly preserved after composite formation. Therefore, the observed morphology variation is attributed to different degrees of NIR association on the ZVO surface rather than reconstruction of the ZVO framework itself. At low NIR content (5 wt.%), the N‐rich component is partially associated with the ZVO aggregates. At the optimized content of 7 wt.%, the NIR is more uniformly distributed around the ZVO particles, maintaining electrolyte‐accessible interfaces. In contrast, excessive NIR incorporation at 10 wt.% may induce local accumulation and partial surface blocking, which can reduce ion‐accessible active sites. The corresponding SEM images are consistent with this interfacial‐decoration model. Pristine ZVO consists of densely packed micron‐scale secondary aggregates assembled from fused primary particles with compact surface features (Figure 3b). After introducing 5 wt.% NIR, the ZVO aggregate morphology is largely retained, while the surface becomes moderately roughened with flake‐like and particulate features, indicating initial association of the N‐rich interfacial component (Figure 3c). For GZVO‐7 wt.%, the aggregates show a roughened and more uniformly decorated surface, suggesting improved contact between ZVO and the NIR framework while preserving accessible interparticle regions (Figure 3d). By contrast, GZVO‐10 wt.% exhibits more pronounced surface accumulation and aggregation, implying that excessive NIR content may reduce electrolyte‐accessible interfaces and hinder Zn^2^^+^ transport pathways (Figure 3e). These SEM observations indicate that GZVO‐7 wt.% provides the most balanced interfacial decoration among the investigated composites, with sufficient NIR association while avoiding excessive surface blocking.

**FIGURE 3:**
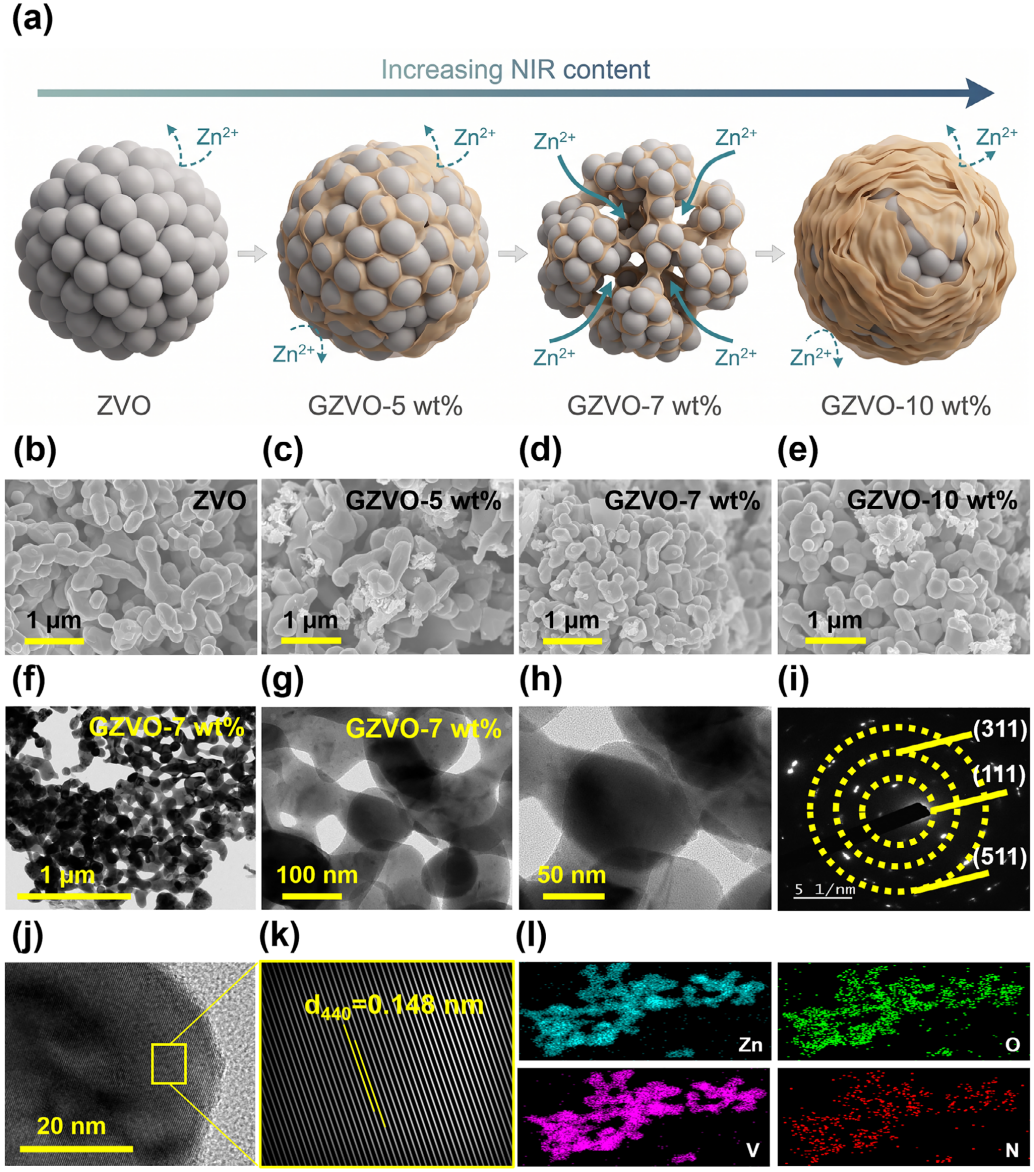
Morphology and microstructure of ZVO and GZVO composites. (a) Schematic illustration of NIR‐content‐dependent morphology evolution. SEM images of (b) ZVO, (c) GZVO‐5 wt.%, (d) GZVO‐7 wt.%, and (e) GZVO‐10 wt.%. (f–h) TEM images, (i) SAED pattern, and (j, k) HR‐TEM images of GZVO‐7 wt.% showing a lattice spacing of 0.148 nm assigned to the (440) plane of spinel ZVO. (l) STEM–EDS elemental maps of Zn, O, V, and N for GZVO‐7 wt.%.

The specific surface areas of ZVO and the GZVO composites were further evaluated using N_2_ adsorption–desorption measurements (Figure S8). The calculated BET specific surface areas of ZVO, GZVO‐5 wt.%, GZVO‐7 wt.%, and GZVO‐10 wt.% are 14.45, 19.68, 24.47, and 8.02 m^2^ g^−1^, respectively. The progressive increase from ZVO to GZVO‐7 wt.% indicates that moderate NIR integration increases the accessible interfacial surface of the composite. In particular, GZVO‐7 wt.% exhibits the highest specific surface area, providing more accessible electrode–electrolyte contact regions and electrochemically available reaction sites. By contrast, the specific surface area decreases substantially to 8.02 m^2^ g^−1^ for GZVO‐10 wt.%. This decrease is consistent with the surface accumulation and aggregation observed by SEM, suggesting that excessive NIR incorporation partially blocks accessible interparticle regions. Therefore, the optimal performance of GZVO‐7 wt.% is attributed to a balanced combination of high accessible surface area, uniform NIR distribution, and favorable interfacial contact rather than to the NIR content alone.

The internal microstructure of the optimized GZVO‐7 wt.% sample was further examined by TEM and high‐resolution TEM (HR‐TEM). The low‐magnification TEM image reveals porous and interconnected aggregates composed of high‐contrast ZVO domains in close contact with lower‐contrast NIR‐associated regions (Figure 3f). Magnified TEM images further show that the crystalline ZVO nanoparticles are interfacially associated with the low‐contrast N‐rich component, without obvious isolated phase segregation (Figure 3g,h). Such an integrated microstructure is favorable for establishing continuous interfacial contact between ZVO and the NIR framework, thereby facilitating electrolyte accessibility and interfacial charge/ion transport. The selected‐area electron diffraction (SAED) pattern of GZVO‐7 wt.% exhibits well‐defined concentric diffraction rings that can be indexed to representative planes of spinel ZVO, including the (111), (311), and (511) reflections (Figure 3i). This result is consistent with the PXRD analysis and confirms that the crystalline spinel ZVO framework is preserved after NIR integration. HR‐TEM images further reveal well‐resolved lattice fringes from the ZVO domains, with an interplanar spacing of 0.148 nm corresponding to the (440) plane of spinel ZVO (Figure 3j,k). Adjacent low‐crystalline regions are observed around the crystalline ZVO domains, supporting the close interfacial association of the NIR component with ZVO. This spatial contact between crystalline ZVO and N‐rich interfacial regions suggests the formation of a structurally integrated composite architecture that can promote interfacial charge redistribution while maintaining the integrity of the spinel host. The elemental composition and spatial distribution of GZVO‐7 wt.% were further analyzed by scanning transmission electron microscopy–energy‐dispersive X‐ray spectroscopy (STEM–EDS) mapping (Figure 3l). The Zn, V, and O signals are uniformly distributed throughout the aggregate, confirming the homogeneous distribution of the ZVO framework. Notably, the N signal is also broadly distributed across the same composite region and overlaps with the Zn/V/O distributions, indicating that the N‐containing interfacial component is well integrated with ZVO rather than segregated into isolated domains. Collectively, the morphological and microstructural results demonstrate that GZVO‐7 wt.% possesses a ZVO/NIR‐integrated architecture with preserved spinel crystallinity, homogeneous elemental distribution, and accessible interfacial contact. This microstructure establishes a structural framework for improved electrolyte accessibility, interfacial charge transport, and Zn^2+^ storage kinetics in the optimized GZVO cathode.

### 2.2 Electrochemical Measurement

The electrochemical performance of ZVO and GZVO‐x (x = 5, 7, 10 wt.%) cathodes was evaluated in AZIBs using Zn metal foil as the anode. A 3 m Zn(CF_3_SO_3_)_2_ aqueous electrolyte was employed to suppress water‐induced side reactions and improve cycling stability in the aqueous environment [17]. The assembled cells were initially discharged to 0.2 V before the first charging process, consistent with the electrochemical sequence used for the ex situ analysis. Unless otherwise specified, all specific capacities were calculated based on the mass of active materials loaded on the carbon cloth current collector, with a typical mass loading of 1.3–1.5 mg cm^−2^. In general, moderate NIR integration is expected to improve interfacial Zn^2+^ accessibility and charge‐transfer kinetics, whereas excessive NIR incorporation may reduce accessible ZVO surface sites and hinder ion transport. Therefore, the following electrochemical results are discussed in terms of the NIR‐content‐dependent balance between interfacial regulation and active‐site accessibility.

The electrochemical redox behavior of ZVO and GZVO composite cathodes was analyzed by cyclic voltammetry (CV). Figure S9 shows the initial CV curves recorded within 0.2–1.8 V *vs*. Zn^2+^/Zn at a scan rate of 0.5 mV s^−1^. For pristine ZVO, two cathodic peaks at approximately 0.507 and 0.869 V and two anodic peaks at about 0.745 and 1.085 V are observed, corresponding to stepwise Zn^2+^ insertion/extraction coupled with vanadium redox activity (Figure S9a). After NIR integration, GZVO‐5 wt.% and GZVO‐7 wt.% electrodes retain the characteristic redox features of ZVO but exhibit sharper and more intense redox peaks, indicating enhanced electrochemical activity and improved interfacial reaction kinetics (Figure S9b, c). In particular, GZVO‐7 wt.% shows the highest peak current and the smallest peak‐position variation during repeated cycles, suggesting superior redox reversibility and reduced polarization. In contrast, GZVO‐10 wt.% displays broader redox peaks and more noticeable potential drift (Figure S9d). The overlaid second‐cycle CV curves in Figure 4a further confirm that GZVO‐7 wt.% has the most favorable redox response among the investigated electrodes.

**FIGURE 4:**
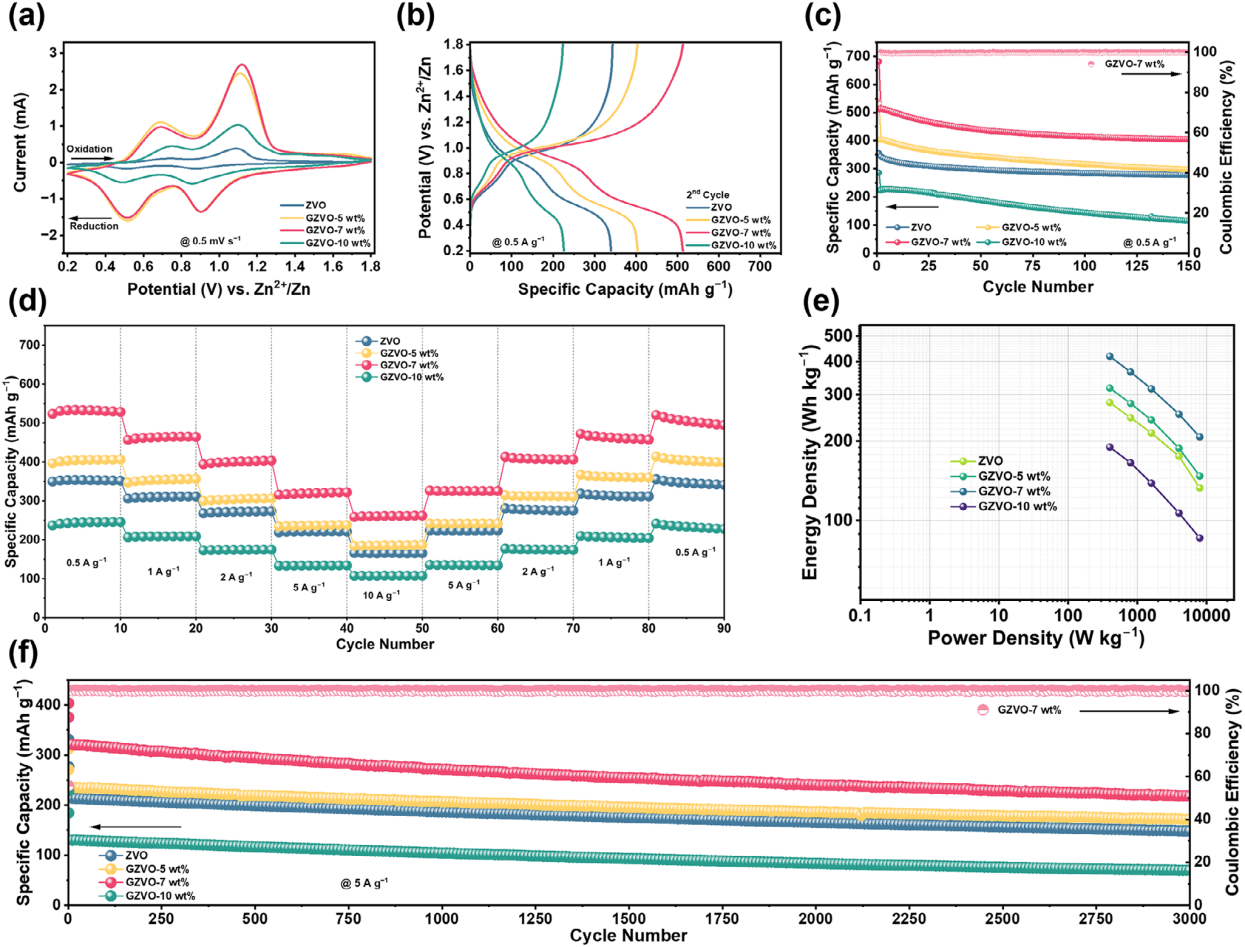
Electrochemical performance of ZVO and GZVO composite cathodes in AZIBs. (a) CV curves at 0.5 mV s^−1^. (b) Galvanostatic charge/discharge profiles at the second cycle under 0.5 A g^−1^. (c) Cycling performance at 0.5 A g^−1^ for 150 cycles. (d) Rate capability at current densities from 0.5 to 10 A g^−1^. (e) Ragone plot comparing energy and power densities. (f) Long‐term cycling performance at 5 A g^−1^ over 3000 cycles.

The galvanostatic charge–discharge (GCD) profiles further support the CV results. Figure S10 presents the initial GCD curves of ZVO and GZVO‐x (x = 5, 7, 10 wt.%) recorded at 0.5 A g^−1^ within 0.2–1.8 V. Pristine ZVO delivers an initial discharge capacity of 344.47 mAh g^−1^, with two discharge plateaus near 0.9 and 0.6 V, consistent with the stepwise Zn^2+^ insertion process (Figure S10a). GZVO‐5 wt.% and GZVO‐7 wt.% deliver higher initial discharge capacities of 403.02 and 511.79 mAh g^−1^, respectively, with more extended discharge plateaus (Figure S10b,c). A notable difference is observed between the first charge and the subsequent charge profiles of the GZVO‐x (x = 5, 7, 10 wt.%) electrodes. The first charge exhibits an extended plateau near 1.0 V, which corresponds to the strong and broadened first‐cycle anodic feature at approximately 1.0–1.1 V in the corresponding dQ/dV profiles (Figure S11). Because the electrochemical measurements begin with discharge to 0.2 V, this plateau is associated with the initial oxidative activation of the discharged electrode. During this stage, Zn^2+^ extraction and vanadium reoxidation occur together with relaxation of discharge‐induced local strain and initial conditioning of the ZVO/GCN/electrolyte interface. The polar N‐rich interface provides heterogeneous near‐surface Zn^2+^ storage environments and promotes interfacial electrolyte access. Therefore, the first charge can additionally involve the release of Zn^2+^ from interfacial sites and the formation or reorganization of an electrolyte‐derived interphase. After this initial conditioning process, the non‐steady contribution decreases substantially, causing the approximately 1.0 V plateau to shorten and producing the closely overlapping second‐to‐fifth‐cycle profiles. The smaller difference between the first and subsequent profiles of pristine ZVO is consistent with its lower contribution from GCN‐derived interfacial activation. Notably, the charge–discharge profiles of GZVO‐7 wt.% from the second to fifth cycles nearly overlap, demonstrating that stable and reversible electrochemical behavior is established after the initial activation cycle. In contrast, GZVO‐10 wt.% delivers a lower discharge capacity of 223.37 mAh g^−1^ with shortened plateaus and greater polarization (Figure S10d). The structural evolution associated with the first‐charge plateau is further examined using the ex situ XRD patterns collected at 0.7, 1.1, and 1.8 V, as discussed in Section 2.4.

Differential capacity (dQ/dV) profiles were further analyzed to support the redox behavior observed from CV and GCD measurements (Figure S11). All electrodes show paired redox peaks corresponding to stepwise Zn^2+^ insertion/extraction, while GZVO‐7 wt.% exhibits sharper peaks with smaller potential shifts during the initial cycles. These results further support the improved redox reversibility and reduced polarization of GZVO‐7 wt.%. To identify the optimized NIR content, the electrochemical performance of ZVO and GZVO‐x (x = 5, 7, 10 wt.%) cathodes was evaluated through cycling stability, rate capability, and long‐term durability tests. Figure 4c compares the cycling performance of the electrodes at 0.5 A g^−1^. All electrodes exhibit an initial activation process, which is commonly observed in vanadium‐based AZIB cathodes and is generally associated with gradual electrolyte infiltration and activation of electrochemically accessible sites [14]. Among the investigated electrodes, GZVO‐7 wt.% delivers the best cycling performance, reaching a discharge capacity of 512.76 mAh g^−1^ at the second cycle and maintaining 405.16 mAh g^−1^ after 150 cycles with a Coulombic efficiency approaching 100%. It should be noted that these capacities are normalized to the total GZVO‐7 wt.% composite active mass, comprising 93 wt.% ZVO and 7 wt.% GCN. Direct comparison with the one‐electron theoretical capacity of pure ZVO gives apparent charge equivalents of 4.42 and 3.50 electrons for 512.76 and 405.16 mAh g^−1^, respectively. These values represent the total measured electrochemical charge and should not be interpreted as literal bulk Zn^2+^‐insertion stoichiometries. The detailed mass‐basis calculation and possible origins of the additional capacity are discussed in Section 2.4. In comparison, pristine ZVO and GZVO‐5 wt.% retain 278.30 and 296.46 mAh g^−1^ after 150 cycles, respectively, whereas GZVO‐10 wt.% delivers only 114.84 mAh g^−1^. These results confirm that 7 wt.% NIR provides the most favorable composition for achieving high reversible capacity and stable cycling performance. To evaluate the electrochemical performance of GZVO‐7 wt.% under increased active‐material loading, electrodes with mass loadings of approximately 1.5, 5, and 10 mg cm^−2^ were cycled at 0.5 A g^−1^ for 100 cycles under identical conditions (Figure S12). Following the initial activation, the electrodes delivered specific capacities of 511.78, 426.48, and 343.63 mAh g^−1^, respectively. After 100 cycles, the corresponding capacities were 415.05, 345.87, and 221.36 mAh g^−1^, representing capacity retentions of 81.10%, 81.10%, and 64.42%, respectively. Notably, the 5 mg cm^−2^ electrode retained the same percentage of its initial capacity as the conventional 1.5 mg cm^−2^ electrode. Although the gravimetric capacity and retention decreased at 10 mg cm^−2^, the electrode still maintained a reversible capacity of 221.36 mAh g^−1^ after 100 cycles. These results confirm that GZVO‐7 wt.% maintains useful cycling performance at elevated mass loadings and identify 5 mg cm^−2^ as the most favorable high‐loading condition.

The rate capability of ZVO and GZVO‐x cathodes was further evaluated by stepwise increasing the current density from 0.5 to 1, 2, 5, and 10 A g^−1^, followed by recovery to 0.5 A g^−1^, as shown in Figure 4d. GZVO‐7 wt.% consistently delivers the highest reversible capacity across the entire current‐density range and maintains 258.93 mAh g^−1^ at 10 A g^−1^. When the current density is returned to 0.5 A g^−1^, the capacity recovers to 520.34 mAh g^−1^, demonstrating excellent rate reversibility and high‐current tolerance. In contrast, ZVO, GZVO‐5 wt.%, and GZVO‐10 wt.% show lower capacities at elevated current densities, indicating less efficient charge‐storage kinetics.

The energy–power characteristics of ZVO and GZVO composite cathodes were evaluated using a Ragone‐type plot, as shown in Figure 4e. The energy density was calculated from the discharge capacity and average discharge voltage, while the power density was estimated from the corresponding current density and voltage output based on the active‐material mass. GZVO‐7 wt.% delivers the highest energy density of 418.83 Wh kg^−1^ at 400 W kg^−1^ and retains 207.14 Wh kg^−1^ at 8000 W kg^−1^. In comparison, ZVO, GZVO‐5 wt.%, and GZVO‐10 wt.% show lower energy densities of 279.57, 317.21, and 189.63 Wh kg^−1^ at 400 W kg^−1^, respectively, which decrease to 132.77, 147.33, and 85.77 Wh kg^−1^ at 8000 W kg^−1^. The superior energy retention of GZVO‐7 wt.% under high‐power operation is consistent with its enhanced rate capability and reduced polarization.

To further evaluate long‐term durability, all electrodes were cycled at 5 A g^−1^ for 3000 cycles, as shown in Figure 4f. GZVO‐7 wt.% exhibits the most stable cycling behavior, maintaining 218.24 mAh g^−1^ after 3000 cycles with a Coulombic efficiency close to 100%. In comparison, ZVO, GZVO‐5 wt.%, and GZVO‐10 wt% deliver lower capacities of 148.42, 171.41, and 70.07 mAh g^−1^, respectively, under identical conditions. These results demonstrate the superior long‐term cycling stability of GZVO‐7 wt.%. The structural stability of the cycled GZVO‐7 wt.% electrode was further examined by ex situ XRD, SEM, and EDS after 1000 and 3000 cycles (Figure S13). The characteristic ZVO‐derived diffraction peaks remain visible after cycling without obvious new crystalline impurity reflections, indicating persistence of a crystalline ZVO‐derived fraction (Figure S13a). SEM images show that the electrode surface remains relatively intact after 1000 cycles and preserves its overall morphology after 3000 cycles, despite slight surface roughening (Figure S13b–e). EDS mapping after 3000 cycles confirms the relatively uniform distribution of Zn, V, O, N, and C across the electrode surface (Figure S13f–j). To examine the GCN/ZVO contact at the nanoscale, additional TEM and STEM–EDS analyses were conducted on GZVO‐7 wt.% recovered after 3000 cycles at 5 A g^−1^. The TEM images show that the nanoscale particle assemblies remain intact without extensive fragmentation or separation in the examined regions (Figure S14a–c). The lattice‐fringe image and spot‐type SAED pattern further confirm the persistence of crystalline domains after prolonged cycling (Figure S14d, e). STEM–EDS mapping shows that Zn, V, and O remain concentrated within the same particle regions, while the N signal remains spatially associated with and distributed around these regions (Figure S14f–j). The retained N distribution demonstrates that the GCN‐derived N‐rich component remains in contact with ZVO after prolonged cycling. These results support the retention of sufficient structural continuity at the physically integrated GCN/ZVO interface, with no evidence of extensive delamination in the examined regions, although localized nanoscale reconstruction cannot be completely excluded. The overall electrochemical performance of GZVO‐7 wt% was further compared with recently reported metal‐oxide/carbon‐composite cathodes for AZIBs, as summarized in Table 2 [28, 29, 38, 39, 40, 41, 42, 43, 44, 45, 46, 47]. GZVO‐7 wt.% maintains 405.16 mAh g^−1^ after 150 cycles at 0.5 A g^−1^, delivers 258.93 mAh g^−1^ at 10 A g^−1^, and retains 218.24 mAh g^−1^ after 3000 cycles at 5 A g^−1^ with 84.3% capacity retention. Its reversible capacity is higher than those reported for Zn_3_V_2_O_7_‐C, Zn_3_V_2_O_7_(OH)_2_·H_2_O@GC, α‐MnO_2_@g‐C_3_N_4_, MnO‐CNT@g‐C_3_N_4_, and ZnMn_2_O_4_@g‐C_3_N_4_ under their respective testing conditions [28, 29, 41, 42, 47]. Furthermore, the capacity of 258.93 mAh g^−1^ at 10 A g^−1^ is close to the 282.54 mAh g^−1^ reported for N‐doped VO_x_@g‐C_3_N_4_ [45] at the same current density and exceeds the high‐rate capacities of the other carbon‐composite cathodes included in Table 2. Together with its stable 3000‐cycle performance, these results demonstrate that GZVO‐7 wt.% provides a highly competitive combination of reversible capacity, high‐rate capability, and long‐term cycling durability. The favorable performance obtained using only 7 wt.% NIR further demonstrates the effectiveness of the optimized NIR‐integrated design for durable and high‐rate vanadium‐based AZIB cathodes.

**TABLE 2:** Comparison of the electrochemical performance of GZVO‐7 wt.% with recently reported metal oxides‐ and carbon‐composite‐based cathode materials for AZIBs.

Material	Method	Electrolyte	Potential window (V)	Specific capacity (mAh g^−1^)	Rate performance (mAh g^−1^)	Retention	Refs.
Zn_3_V_2_O_7_(OH)_2_·2H_2_O	Microwave	1 m ZnSO_4_	0.2–1.8	213 at 0.05 A g^−1^	54 at 3 A g^−1^	68% at 0.2 A g^−1^	[38]
Zn_3_V_2_O_7_(OH)_2_·2H_2_O	Hydrothermal	3 m Zn(CF_3_SO_3_)_2_	0.2–1.6	237.7 at 0.5 A g^−1^	145.9 at 1 A g^−1^	96.7 at 1 A g^−1^	[39]
ZnV_6_O_16_·8H_2_O	Ion‐exchange	2 m ZnSO_4_	0.25–1.6	365 at 0.5 A g^−1^	167 at 15 A g^−1^	70% at 3 A g^−1^	[40]
Zn_3_V_2_O_7_‐C	Co‐precipitation	2.5 m Zn(CF_3_SO_3_)_2_	0.2–1.6	430 at 0.1 A g^−1^	210 at 0.5 A g^−1^		[41]
Zn_3_V_2_O_7_(OH)_2_·H_2_O@GC	Hydrothermal	2 m Zn(OTF)_2_	0.5–1.8	166 at 0.1 A g^−1^	58 at 5 A g^−1^		[42]
Zn_2_V_2_O_7_	Solvothermal	3 m Zn(CF_3_SO_3_)_2_	0.5–1.75	329.8 at 0.1 A g^−1^	271.7 at 10 A g^−1^	91.3% at 10 A g^−1^	[43]
Zn_0.1_V_2_O_5_·nH_2_O Xerogel	Hydrothermal	2 m ZnSO_4_	0.25–1.7	432 at 0.2 A g^−1^	280 at 6 A g^−1^	88% at 10 A g^−1^	[44]
N‐doped VO_x_@g‐C_3_N_4_	Hydrothermal	3 m Zn(CF_3_SO_3_)_2_	0.2–1.6	500.18 at 0.2 A g^−1^	282.54 at 10 A g^−1^	87.33% at 10 A g^−1^	[45]
α ‐MnO_2_@g‐C_3_N_4_	Hydrothermal	2 m ZnSO_4_	0.8–2.0	298 at 0.1 A g^−1^	121 at 2 A g^−1^	83.4% at 1 A g^−1^	[28]
K^+^/C_3_N_4_ co‐intercalated NH_4_V_4_O_10_	Hydrothermal	3 m Zn(CF_3_SO_3_)_2_	0.2–1.6	348.5 at 2 A g^−1^	228.4 at 20 A g^−1^	78.2% at 10 A g^−1^	[46]
MnO‐CNT@C_3_N_4_	Hydrothermal	2 m ZnSO_4_ + 0.2 m MnSO_4_	0.8–1.8	209 at 0.8 A g^−1^	101 at 3 A g^−1^		[47]
ZnMn_2_O_4_@g‐C_3_N_4_	Solvothermal	2 m ZnSO_4_ + 0.2 m MnSO_4_	0.8–1.8	142.9 at 0.3 A g^−1^	26.9 at 2 A g^−1^	75.1% at 1 A g^−1^	[29]
N‐rich interfacial regulator‐integrated ZnV_2_O_4_	ultrasonication‐assisted physical integration	3 m Zn(CF_3_SO_3_)_2_	0.2–1.8	405 at 0.5 A g^−1^	258.93 at 10 A g^−1^	84.3% at 5 A g^−1^	This Work

### 2.3 Zn^2+^ Storage Kinetic Properties

To elucidate the Zn^2+^ storage kinetics of the electrodes, CV measurements were conducted at scan rates from 0.2 to 1.0 mV s^−1^, as shown in Figure 5a,b and Figures S15a and S16a. All electrodes exhibit distinguishable redox peaks associated with reversible Zn^2+^ storage. Among them, GZVO‐7 wt.% shows the largest enclosed CV area and the highest current response over the entire scan‐rate range, indicating enhanced electrochemical activity and faster redox kinetics.

**FIGURE 5:**
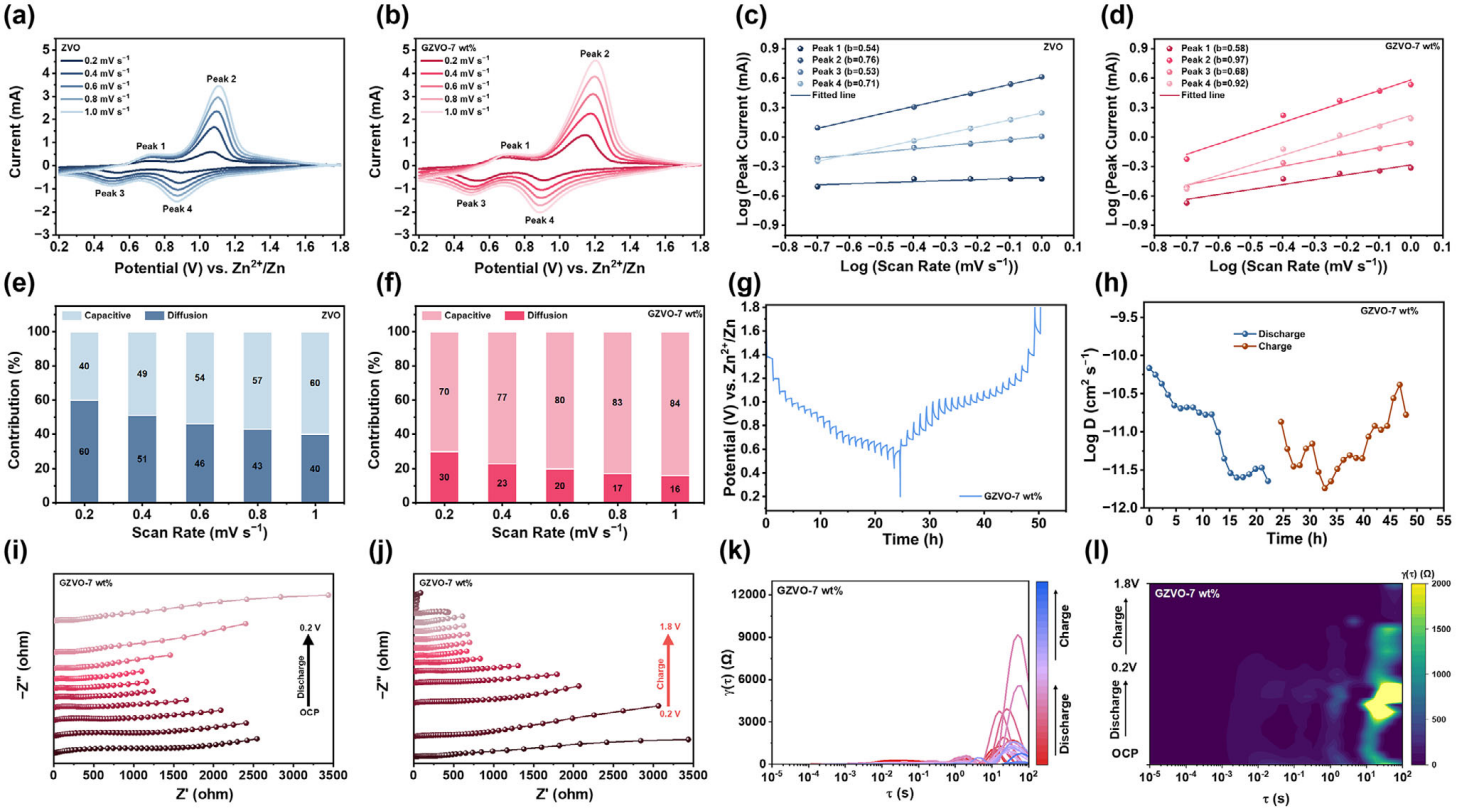
Zn^2+^ storage kinetics of ZVO and GZVO‐7 wt.% cathodes. CV curves at different scan rates for (a) ZVO and (b) GZVO‐7 wt.%. Corresponding *b*‐value fitting plots of redox peaks for (c) ZVO and (d) GZVO‐7 wt.%. Capacitive and diffusion‐controlled contribution ratios at various scan rates for (e) ZVO and (f) GZVO‐7 wt.%. (g) GITT profile, (h) calculated DZn^2+^ of GZVO‐7 wt.%. (i, j) In situ EIS evolution of GZVO‐7 wt.% during discharge and charge. (k, l) DRT profiles and corresponding 2D DRT contour map of GZVO‐7 wt.%.

The kinetic characteristics were further analyzed using the power‐law relationship(i = av^b^), where i is the peak current, v is the scan rate, and b reflects the charge‐storage behavior. A *b*‐value close to 0.5 indicates a diffusion‐controlled kinetics, whereas a value approaching 1.0 suggests a surface‐controlled capacitive behavior. As shown in Figure 5c, pristine ZVO exhibits *b*‐values of 0.54, 0.76, 0.53, and 0.71 for Peaks 1–4, respectively, indicating mixed diffusion‐controlled and surface‐controlled kinetics. After NIR integration, the *b*‐values increase, especially for GZVO‐7 wt.%, which delivers values of 0.58, 0.97, 0.62, and 0.92 for Peaks 1–4, respectively (Figure 5d). The near‐unity *b*‐values for Peaks 2 and 4 reveal a strong pseudocapacitive contribution, indicating that the optimized NIR‐integrated architecture effectively accelerates surface‐dominated redox kinetics. By comparison, GZVO‐5 wt.% (Figure S15b) and GZVO‐10 wt.% (Figure S16b) show relatively lower b‐values, suggesting less efficient kinetic enhancement.

To quantitatively distinguish capacitive and diffusion‐controlled contributions, Dunn's method was applied according to: i(V) = k_1_v + k_2_v^0.5^, where k_1_v and k_2_v^0.5^ represent the capacitive and diffusion‐controlled components, respectively. As shown in Figure 5e, the capacitive contribution of pristine ZVO increases from 40% at 0.2 mV s^−1^ to 60% at 1.0 mV s^−1^, while GZVO‐5 wt.% shows a moderate increase from 43% to 63% (Figure S15c). Notably, GZVO‐7 wt.% exhibits much higher capacitive contributions of 70%, 77%, 80%, 83%, and 84% at scan rates of 0.2, 0.4, 0.6, 0.8, and 1.0 mV s^−1^, respectively, exceeding those of the other electrodes (Figure 5f). This dominant capacitive contribution indicates faster interfacial charge transfer and reduced Zn^2+^ diffusion limitations. In contrast, the lower capacitive contribution of GZVO‐10 wt.% confirms that excessive NIR incorporation does not further improve the kinetic response and may instead hinder active‐site accessibility (Figure S16c). The superior kinetic behavior of GZVO‐7 wt.% can be attributed to its optimized NIR‐integrated architecture, which provides accessible interfacial sites, promotes charge redistribution, and facilitates Zn^2+^ transport across the electrode/electrolyte interface. Consequently, GZVO‐7 wt.% exhibits enhanced pseudocapacitive behavior, faster redox kinetics, and improved Zn^2+^ storage reversibility, consistent with its superior rate capability and cycling stability. These kinetic analyses demonstrate that 7 wt.% NIR effectively shifts the Zn^2+^ storage process toward a more surface‐dominated and highly reversible reaction pathway.

Galvanostatic intermittent titration technique (GITT) measurements were performed to evaluate the apparent Zn^2+^ transport behavior of GZVO composite electrodes. The measurements were conducted at 0.1 A g^−1^ using a 10 min current pulse followed by a 1 h relaxation period. All the electrodes exhibit staircase‐like discharge/charge profiles, consistent with reversible Zn^2+^ insertion/extraction behavior. The potential relaxation after each current pulse reflects the combined effects of interfacial polarization and Zn^2+^ diffusion within the electrode. Compared with GZVO‐5 wt.% and GZVO‐10 wt.%, GZVO‐7 wt.% shows a more regular potential evolution and smaller voltage polarization during discharge/charge indicating improved reaction reversibility and lower overall polarization (Figure 5g). The larger polarization observed for GZVO‐5 wt.% (Figure S17a) suggests insufficient interfacial optimization, whereas the pronounced potential fluctuation of GZVO‐10 wt.% (Figure S18a) indicates that excessive NIR incorporation may restrict electrolyte accessibility and partially hinder ion‐accessible active sites.

The corresponding apparent Zn^2+^ diffusion coefficients calculated from the GITT profiles are shown in Figure 5h and Figures S17b and S18b. The DZn^2+^ values of GZVO‐5 wt.%, GZVO‐7 wt.%, and GZVO‐10 wt.% are in the range of 3.34 × 10^−12^–7.94 × 10^−11^, 1.82 × 10^−12^–4.06 × 10^−11^, and 3.93 × 10^−12^–7.08 × 10^−11^ cm^2^ s^−1^, respectively. These values fall within the typical diffusion range reported for vanadate‐based AZIB cathodes, confirming feasible Zn^2+^ migration in all composite electrodes [48]. Therefore, GZVO‐7 wt.% does not show the highest apparent diffusion coefficient among the composites. Its superior rate performance is instead attributed mainly to reduced polarization and surface‐dominated Zn^2+^ storage, as supported by the high *b*‐values and large capacitive contribution discussed above. Thus, the GITT results indicate that GZVO‐7 wt.% enables more reversible Zn^2+^ storage with lower polarization, rather than simply faster bulk Zn^2+^ diffusion.

To further examine interfacial reaction kinetics, in situ electrochemical impedance spectroscopy (EIS) combined with distribution of relaxation times (DRT) analysis was conducted during the discharge/charge process. DRT analysis deconvolutes overlapping impedance responses into distinguishable relaxation processes, providing insight into electrolyte transport, interfacial charge transfer, and diffusion‐associated polarization [49, 50]. During discharge, the Nyquist plots of all electrodes show a general decrease in impedance from the open‐circuit potential state to intermediate discharge states, indicating gradual electrode activation and improved electrolyte penetration. Pristine ZVO maintains relatively large semicircles, reflecting sluggish interfacial charge transfer (Figure S19a). GZVO‐5 wt.% shows partially reduced impedance (Figure S20a), while GZVO‐7 wt.% exhibits the smallest impedance evolution and lowest polarization over the monitored discharge region (Figure 5i). GZVO‐10 wt.% displays increased impedance compared with GZVO‐7 wt.%, suggesting less favorable interfacial kinetics (Figure S21a). During the subsequent charge process, the impedance response gradually evolves as Zn^2+^ ions are extracted from the host framework (Figure 5j and Figures S19b, S20b and S21b). Among all electrodes, GZVO‐7 wt.% retains the lowest polarization, indicating more reversible interfacial reaction behavior.

The corresponding DRT profiles further clarify the kinetic differences among the electrodes. Pristine ZVO shows intense relaxation peaks related to interfacial charge‐transfer and diffusion‐associated polarization, indicating high kinetic resistance (Figure S19c, d). The relaxation intensity decreases for GZVO‐5 wt.% (Figure S20c, d), while GZVO‐7 wt.% exhibits the weakest relaxation intensity and the most compact resistance distribution across the monitored state‐of‐charge range (Figure 5k,l). In contrast, GZVO‐10 wt.% shows broadened relaxation features, especially in the intermediate‐ and low‐frequency regions (Figure S21c, d). These results indicate that GZVO‐7 wt.% has the lowest interfacial polarization and most favorable charge‐transfer response among the investigated electrodes. Together with the high capacitive contribution and rate performance, the EIS–DRT results support the surface‐dominated fast charge‐storage behavior of GZVO‐7.

### 2.4 Zn^2+^ Storage Mechanism

The Zn^2+^ storage mechanism of the optimized GZVO‐7 wt.% cathode was investigated by ex situ XRD and XPS at selected electrochemical states, including the pristine electrode, fully discharged state at 0.2 V, and fully charged state at 1.8 V. Before discussing the structural evolution, the measured capacity was correlated with the apparent charge‐storage stoichiometry. ZnV_2_O_4_ has a molar mass of 231.26 g mol^−1^. Therefore, the theoretical capacity corresponding to one electron per ZVO formula unit is calculated as follows:

$$Q=F/\left(3.6M\right)=96485/\left(3.6\times 231.26\right)=115.9\;\mathrm{mAh}\;g^{-1}.$$

All GZVO‐7 capacities are normalized to the total composite mass, comprising 93 wt.% ZVO and 7 wt.% GCN. Direct division of the measured capacities by 115.9 mAh g^−1^ therefore gives composite‐basis apparent electron equivalents of 4.42 for the second‐cycle capacity of 512.76 mAh g^−1^ and 3.50 for the capacity of 405.16 mAh g^−1^ after 150 cycles. If all measured charge is provisionally assigned to the 0.93 ZVO mass fraction, the corresponding upper‐bound ZVO‐equivalent values are 4.76 and 3.76 electrons per ZnV_2_O_4_ formula unit, respectively.

For a formal Zn^2+^‐insertion reaction,

$$ZnV_{2}O_{4}+xZn^{2+}+2xe^{-}⇌Zn_{1+x}V_{2}O_{4}$$

the apparent 4.42‐electron value would correspond to x = 2.21 Zn^2+^ per ZVO. Such a bulk insertion stoichiometry is not established by the present data. Therefore, neither the 4.42‐electron composite‐basis value nor the 4.76‐electron ZVO‐corrected upper bound should be treated as a literal electron count associated exclusively with Zn^2+^ insertion into an unchanged spinel lattice. The high second‐cycle capacity more reasonably reflects the combined contributions of electrochemical activation or reconstruction, possible Zn^2+^/H^+^ co‐storage, surface‐controlled and pseudocapacitive vanadium redox, and electrolyte‐derived interphase reactions [12, 26, 51, 52]. Contributions from the GCN‐containing interface may also be present but cannot be separated quantitatively from the galvanostatic capacity. Determination of the actual Zn^2+^/H^+^ storage stoichiometry would require state‐resolved elemental and mass‐balance measurements together with operando structural or spectroscopic analysis.

Figure 6a presents a nine‐state ex situ XRD sequence comprising the pristine electrode, first discharge to 0.2 V, first charge to 0.7, 1.1, and 1.8 V, second discharge to 0.9, 0.5, and 0.2 V, and second charge to 1.8 V. Importantly, the pattern collected at 1.1 V during the first charge directly probes the structural state of GZVO‐7 wt.% within the extended approximately 1.0 V charge plateau observed in Figure S10c. Because the electrochemical measurement begins with discharge to 0.2 V, this plateau represents an anodic process involving initial oxidative activation superimposed on Zn^2+^ extraction and vanadium reoxidation. The strong and broadened first‐cycle anodic feature at approximately 1.0–1.1 V in the corresponding dQ/dV profile (Figure S11c), followed by its reduction and stabilization in later cycles, further supports this interpretation. The dominant ZVO‐derived reflections remain discernible throughout these states, indicating that a crystalline ZVO fraction persists during the initial two discharge‐charge cycles. The (311) reflection shifts slightly from approximately 35.50° in the pristine state to 35.45° after the first discharge and returns to approximately 35.48° after charging to 1.8 V (Figure 6b). This partly reversible variation is consistent with lattice strain and partial structural recovery associated with ion uptake and release. At the intermediate first‐charge state of 1.1 V, the dominant ZVO‐derived reflections remain visible, and no new well‐resolved crystalline diffraction peaks are detected. Therefore, the extended first‐charge plateau is not associated with complete collapse of the ZVO‐derived crystalline framework or extensive formation of a new highly crystalline phase. Instead, the limited peak‐position and relative‐intensity variations are consistent with Zn^2+^ extraction, vanadium oxidation, relaxation of discharge‐induced lattice strain, and local or poorly crystalline interfacial reorganization during the initial activation process. The more pronounced first‐charge plateau in the GZVO‐x (x = 5, 7, 10 wt.%) electrodes than in pristine ZVO is consistent with an additional conditioning contribution from the polar GCN/ZVO/electrolyte interface. Once this non‐steady interfacial and local structural adjustment is largely completed, its contribution decreases, explaining the substantial shortening of the approximately 1.0 V plateau and the improved coincidence of the second and subsequent charge–discharge profiles. Nevertheless, persistence of the ZVO‐derived reflections should not be interpreted as complete preservation of the initial spinel phase. The marked changes in relative diffraction intensity and incomplete recovery suggest that retained crystalline ZVO domains may coexist with electrochemical activation and local structural reorganization. Chen et al. reported high‐rate stability for a carbon‐integrated accordion‐like ZVO cathode [51], whereas Zhang et al. subsequently demonstrated irreversible first‐cycle formation of ZnV_3_O_8_ and Zn_3_(OH)_2_V_2_O_7_·2H_2_O from a related ZVO@C cathode [26]. Furthermore, although the recent study concerns VO_2_(B) rather than ZnV_2_O_4_, its observation of current‐density‐dependent interphase formation and V^4+^/V^5+^ activation emphasizes the condition‐dependent structural and interfacial evolution of vanadium‐based cathodes [52]. No unambiguous additional sharp reflections assignable to ZnV_3_O_8_ or Zn_3_(OH)_2_V_2_O_7_·2H_2_O are resolved in the present patterns above the detection limit of laboratory XRD. However, amorphous, nanocrystalline, transient, surface‐confined, or low‐fraction reconstructed phases cannot be excluded. Therefore, the present results support persistence and partial recovery of ZVO‐derived crystalline domains rather than complete retention of an unchanged spinel framework. The storage mechanism is consequently interpreted as involving a retained crystalline ZVO fraction together with possible first‐cycle activation, surface reconstruction, vanadium redox, Zn^2+^/H^+^‐coupled storage, and dynamic interphase formation.

**FIGURE 6:**
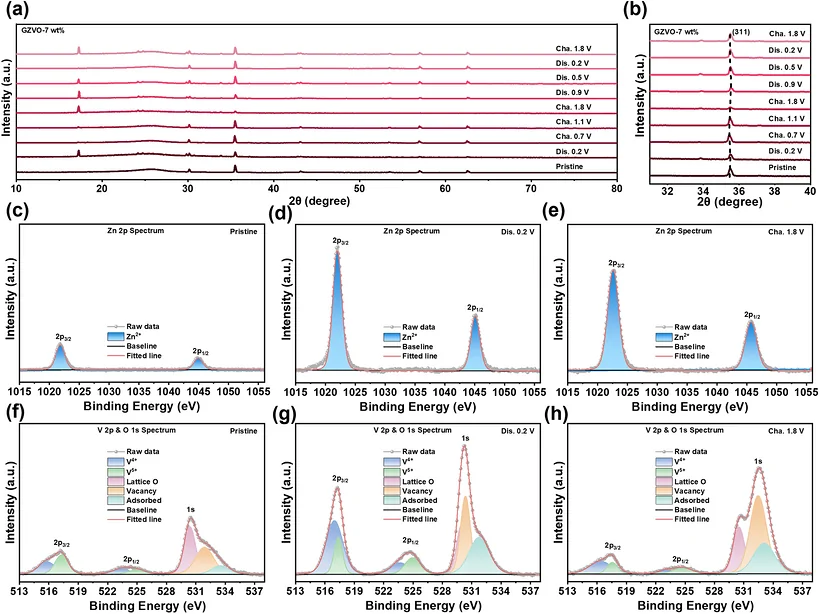
Ex situ structural and chemical‐state analysis of the GZVO‐7 wt.% cathode at different charge/discharge states. (a) Ex situ XRD patterns collected from the pristine electrode and selected discharge/charge states. (b) Enlarged XRD patterns around the (311) reflection, showing reversible peak variation during Zn^2+^ insertion/extraction. High‐resolution ex situ XPS spectra of (c–e) Zn 2p and (f–h) V 2p/O 1s for pristine, fully discharged at 0.2 V, and fully charged at 1.8 V of GZVO‐7 wt.% electrodes.

The ex situ XPS results further elucidate the surface redox evolution and interfacial chemical regulation of the optimized GZVO‐7 wt.% cathode during Zn^2+^ storage. The survey spectra confirm the coexistence of Zn, V, O, C, and N in the pristine, fully discharged, and fully charged electrodes, indicating that the NIR‐integrated surface chemistry is retained during electrochemical cycling (Figure S22). In the Zn 2p spectra (Figure 6c–e), the Zn^2+^ doublet is preserved at all selected states, while the intensified Zn signal after discharge indicates Zn^2+^ uptake into/onto the cathode. The V 2p spectra reveal clear surface valence‐state evolution during cycling (Figure 6f–h). In the pristine electrode, the XPS detected V^5+^ and V^4+^ fractions are 54.32% and 45.68%, respectively. After discharge to 0.2 V, the V^5+^ fraction decreases to 25.89%, whereas the V^4+^ fraction increases to 74.11%, indicating the reduction of surface vanadium species during Zn^2+^ insertion. Upon charging to 1.8 V, the V^5+^ fraction partially recovers to 30.09%, indicating reversible but incomplete surface reoxidation. This surface V^5+^/V^4+^ evolution supports the participation of interfacial vanadium redox in the Zn^2+^ storage process. Considering the high capacitive contribution discussed in the above sections, the overall capacity is attributed to combined Zn^2+^ insertion/extraction, surface/interfacial vanadium redox, and pseudocapacitive charge storage rather than bulk Zn^2+^ intercalation alone.

The O 1s spectra provide further insight into the local oxygen environment during discharge/charge. After discharge, the lattice‐oxygen contribution decreases, while the adsorbed oxygen‐containing component increases, suggesting enhanced interaction with inserted/adsorbed Zn^2+^ and electrolyte‐derived surface species. After charging, partial recovery and reorganization of the oxygen‐related components are observed. Because XPS is surface‐sensitive, these changes are assigned to dynamic surface oxygen environments rather than direct quantification of bulk oxygen vacancies. To identify the influence of ZVO integration on the nitrogen environments of GCN, the high‐resolution N 1s spectrum of pristine GCN was measured as a reference (Figure S6) and compared with that of GZVO‐7 wt.% (Figure S23). Pristine GCN exhibits four fitted components at 399.29, 400.98, 401.66, and 404.94 eV, corresponding to sp_2_‐hybridized C═N─C nitrogen, tertiary N─(C)_3_, terminal/amino C─N─H species, and a *π–π* ^*^ excitation or shake‐up satellite, respectively. In GZVO‐7 wt.%, the corresponding C═N─C, N─(C)_3_, and C─N─H components occur at 398.59, 400.10, and 401.07 eV, representing shifts of approximately −0.70, −0.88, and −0.59 eV relative to pristine GCN. The coherent shifts of the three nitrogen environments indicate electronic perturbation of the GCN framework after interfacial association with ZVO and are consistent with interfacial charge redistribution. Importantly, because the component near 400–401 eV is already present in pristine GCN, it is retained as intrinsic tertiary N─(C)_3_ and is not regarded as evidence for the formation of a new covalent Zn–N phase. Instead, the electronically perturbed and polar nitrogen environments are proposed to facilitate dynamic adsorption and coordination‐like interactions with Zn^2+^ during electrochemical cycling. Here, Zn^2^^+^···N denotes a dynamic Lewis acid–base interaction at the electrode/electrolyte interface rather than the formation of permanent covalent Zn─N or V─N bonds between GCN and ZVO. To examine whether the intrinsic nitrogen environments respond to electrochemical Zn^2+^ storage, the N 1s spectra of pristine, fully discharged, and fully charged GZVO‐7 wt.% electrodes were further compared (Figure S23). In all three states, the fitted components were consistently assigned to C═N─C, tertiary N─(C)_3_, and terminal/amino C─N─H species. The relative contribution of the tertiary N─(C)_3_ component changes from 47.61% in the pristine electrode to 58.98% in the fully discharged state and subsequently to 6.04% in the fully charged state. The increase during discharge is consistent with enhanced electronic perturbation of the polar tertiary nitrogen environment during Zn^2+^ uptake, whereas its pronounced decrease after charging indicates weakening or reorganization of this interaction following Zn^2+^ extraction. Because XPS peak areas are relative and can also be influenced by surface coverage, electrolyte‐derived interphase species, and attenuation effects, these values are not interpreted as absolute fractions of Zn‐coordinated nitrogen. Nevertheless, the state‐dependent redistribution of the intrinsic N 1s components demonstrates that the GCN nitrogen environment is electrochemically responsive and supports its active role in regulating interfacial Zn^2+^ adsorption, charge redistribution, and transport. The C 1s spectra further support the stability of the N‐rich framework and the formation of a dynamic cathode–electrolyte interphase (Figure S24). The variation in the survey C 1s peak intensity shown in Figure S22 should not be interpreted directly as a change in the amount of GCN. The electrode contains several carbon sources, including the GCN component, Super P conductive carbon, PVDF binder, the carbon‐cloth substrate, adventitious surface carbon, and electrolyte‐ or interphase‐derived carbon species. Based on the 70:20:10 electrode composition and 7 wt.% GCN content in GZVO‐7 wt.%, GCN constitutes only 4.9 wt.% of the total dry coating. In addition, because XPS is surface‐sensitive, the survey‐peak intensity is strongly influenced by sampling position, surface coverage, signal attenuation, and spectral normalization. After discharge to 0.2 V, the high‐resolution C 1s spectrum exhibits additional CF_3_/CF_x_‐containing components and attenuation of the relative C─N/N─C═N contribution. These fluorinated signals are consistent with state‐dependent coverage by Zn(CF_3_SO_3_)_2_‐derived interfacial species together with possible contributions from the PVDF‐containing electrode environment. Therefore, they support the formation or reorganization of a dynamic cathode–electrolyte interphase but are not considered independent proof of electrolyte decomposition. After charging to 1.8 V, the principal carbon‐nitride‐related components largely recover, while the fluorinated high‐binding‐energy contribution decreases substantially, indicating partial reversibility of the surface interphase. The recovery of the C─N‐containing spectral envelope, together with the continued presence of the N 1s signal, does not support irreversible degradation or removal of the GCN framework during the examined discharge–charge cycle. These results also clarify that the carbon component is not a completely inert backbone. The conjugated carbon skeleton provides a structural and electronic scaffold for distributing the polar nitrogen sites, maintaining the ZVO/GCN interface, and accommodating dynamic interphase formation. However, no separate carbon‐centered redox process can be established from the present C 1s spectra. The specific interaction with Zn^2+^ is therefore considered to be dominated by the polar nitrogen environments, while the carbon framework provides a complementary structural, interfacial, and charge‐redistribution role. Detailed O 1s, N 1s, and C 1s fitting ratios are provided in Table S2. Collectively, the ex situ XRD and XPS results reveal that Zn^2+^ storage in GZVO‐7 wt.% involves reversible Zn^2+^ insertion/extraction associated with a persistent ZVO‐derived crystalline fraction, surface V^5+^/V^4+^ redox evolution, local oxygen‐environment modulation, reversible N‐site‐assisted Zn^2+^ coordination, and dynamic interphase formation. This integrated storage mechanism explains the high electrochemical reversibility, accelerated reaction kinetics, and long‐term cycling durability of the optimized GZVO‐7 wt.% cathode.

Based on the ex situ structural and surface‐chemical analyses, Figure 7 schematically summarizes the proposed Zn^2+^ storage mechanism of the optimized GZVO‐7 wt.% cathode. During discharge, Zn^2+^ insertion/adsorption occurs together with surface V^5+^/V^4+^ redox evolution and local oxygen‐environment adjustment. Meanwhile, polar N sites in the NIR framework interact with Zn^2+^, facilitating interfacial Zn^2+^ adsorption/migration and charge transfer. During charge, Zn^2+^ extraction is accompanied by partial vanadium reoxidation, weakened Zn^2+^–N interaction, and interphase reorganization. The retained ZVO‐derived diffraction features indicate persistence of a crystalline ZVO fraction during this process. Therefore, Zn^2+^ storage in GZVO‐7 wt.% proceeds through a reversible insertion/extraction process coupled with surface redox, N‐site‐assisted interfacial regulation, and dynamic surface‐environment modulation.

**FIGURE 7:**
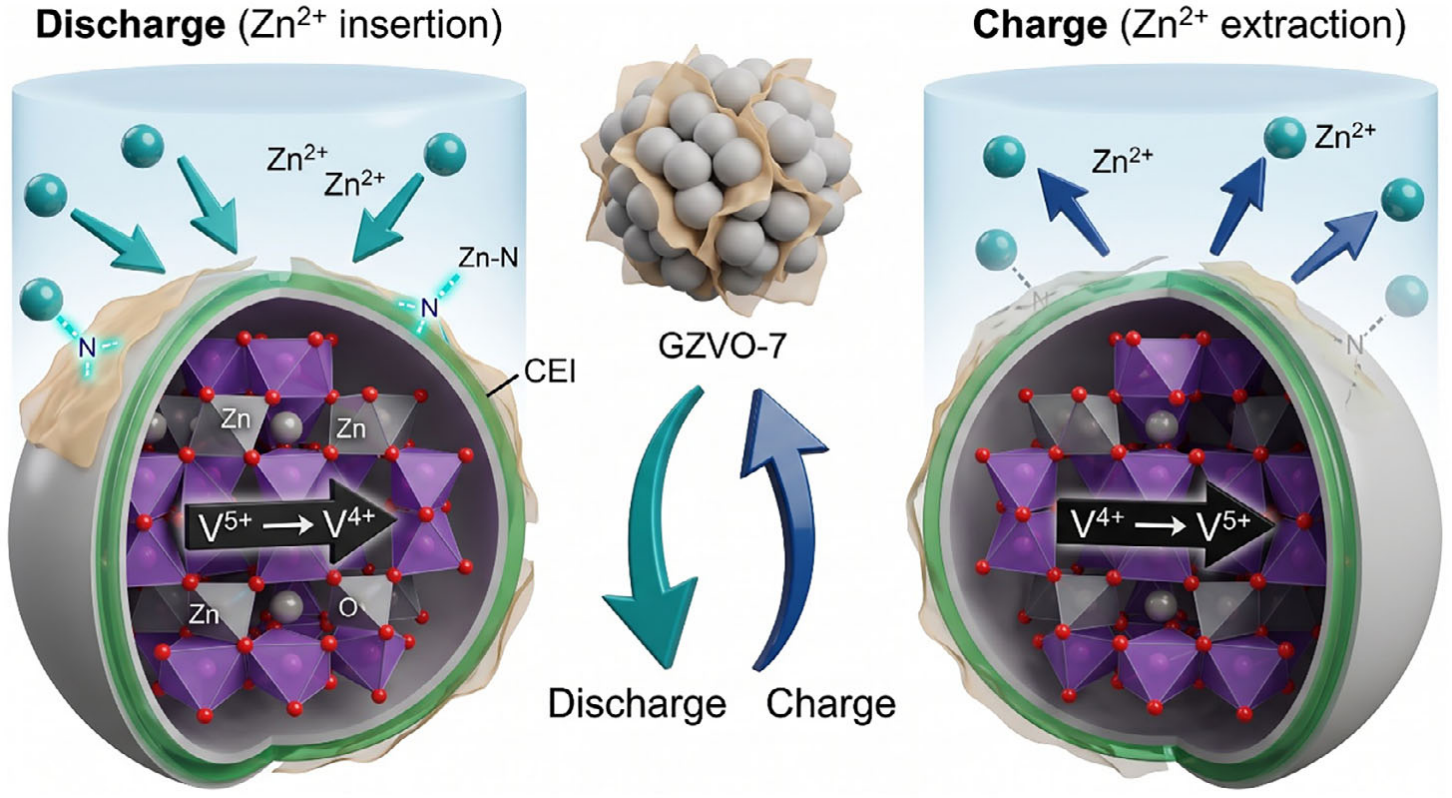
Zn^2+^ storage mechanism of the NIR‐integrated GZVO‐7 wt.% cathode during discharge and charge, involving reversible Zn^2+^ insertion/extraction, V^5+^/V^4+^ redox evolution, N‐site‐assisted Zn^2+^ regulation, oxygen‐environment modulation, dynamic cathode/electrolyte interphase evolution, and persistence of ZVO‐derived crystalline domains.

### 2.5 Density Functional Theory Calculations

Density functional theory (DFT) calculations were performed to gain atomic‐level insight into the interfacial Zn^2+^ energetics and structural stability of the NIR‐integrated GZVO interface compared with pristine ZVO. As shown in Figure 8a, a ZVO (111) slab was constructed as a representative low‐index surface of the spinel framework. To model the interfacial configuration of GZVO, a heptazine unit was introduced onto the ZVO surface as a simplified structural motif of the NIR (Figure 8b). This model enables evaluation of the local interaction between the N‐rich interfacial component and the ZVO surface. It should be noted that the experimental ZVO material exhibits a Zn‐rich elemental composition, whereas the computational slab represents an ideal stoichiometric ZVO crystalline domain. Furthermore, the single heptazine unit is a simplified representation of the extended polymeric GCN structure. Therefore, the present calculations are intended to provide comparative insight into the local effect of a representative N‐rich motif on the ZVO like crystalline surface rather than a complete atomistic description of all Zn‐containing environments or the structurally complex experimental interface. The relative surface Zn‐removal energy was first calculated to evaluate the energetic feasibility of Zn release from the electrode surface. As shown in Figure 8c, the NIR‐integrated GZVO model exhibits a more favorable Zn‐removal energy of −3.4 eV compared with −2.4 eV for pristine ZVO. This result suggests that the N‐rich interfacial environment facilitates surface Zn removal rather than strongly immobilizing Zn species at the ZVO surface. Such energetically favorable Zn release is consistent with the experimentally observed reduced polarization, enhanced rate capability, and improved Zn^2+^ storage kinetics of the GZVO electrode.

**FIGURE 8:**
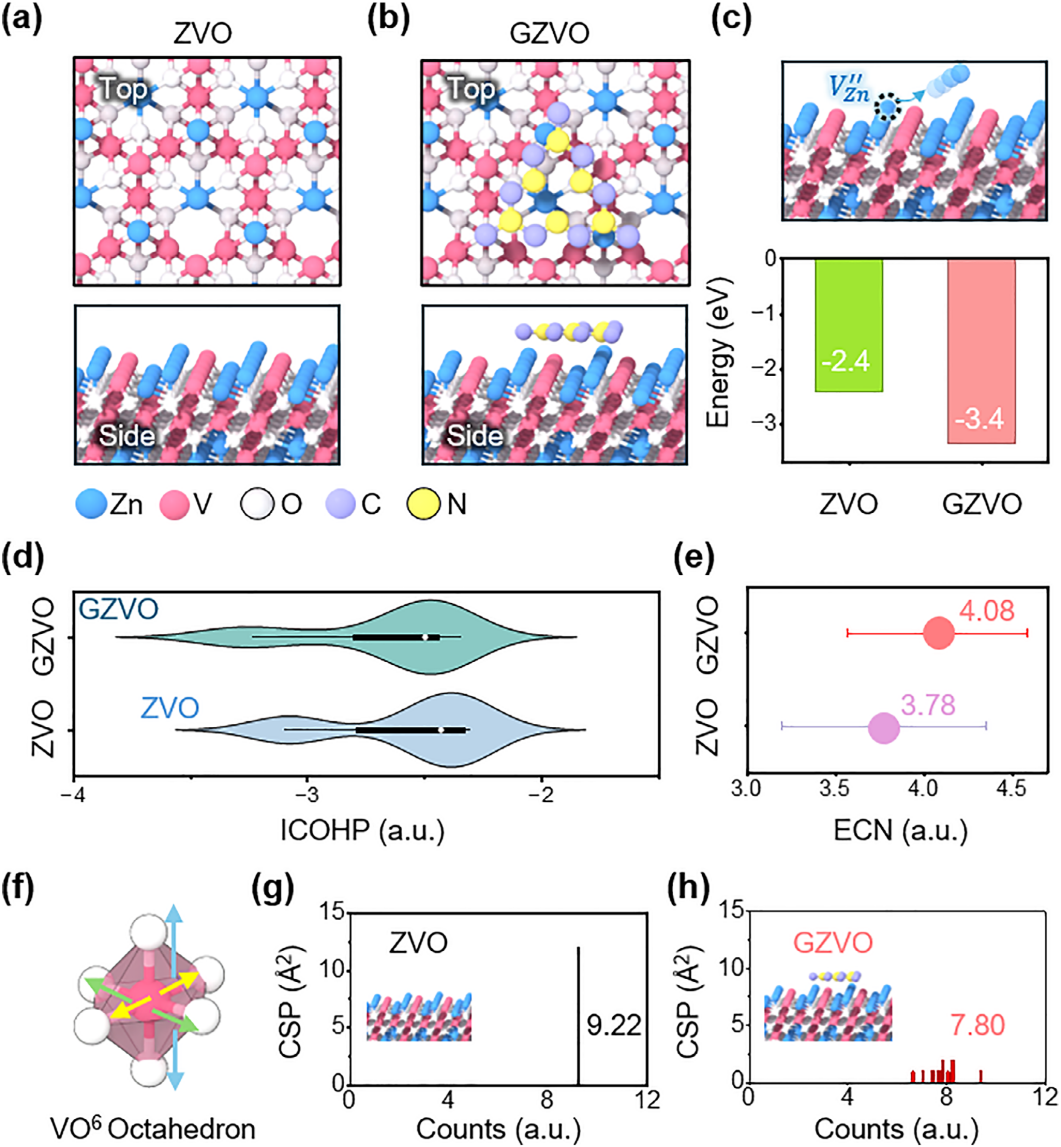
DFT analysis of Zn^2+^ transport and structural stability in ZVO and GZVO. (a, b) Top and side views of the ZVO surface and GCN‐modified GZVO interfacial models. (c) Relative surface Zn‐removal energy of ZVO and GZVO. (d) ICOHP distribution of V─O bonds. (e) Effective coordination number (ECN) of V─O coordination environments. (f) Schematic illustration of the centrosymmetry parameter (CSP) analysis. (g, h) CSP evaluation of ZVO and GZVO, showing reduced local structural distortion after GCN modification.

The structural stability of the spinel host was further examined by analyzing the local V─O coordination environment, because preservation of the VO_6_ framework is essential for reversible Zn^2+^ insertion/extraction. Integrated crystal orbital Hamilton population (ICOHP) analysis was employed to assess the V─O bonding strength (Figure 8d). The more negative average ICOHP value of the GZVO model compared with pristine ZVO indicates strengthened V─O interactions after NIR integration. This reinforced V─O bonding is beneficial for maintaining the structural integrity of the vanadium–oxygen framework during repeated Zn^2+^ uptake/release.

To further quantify the local coordination environment, the effective coordination number (ECN) of V–O was calculated using:

$$\mathrm{ECN}=\sum_{i}\exp(1-{\left(\frac{l_{i}}{l_{\min}}\right)}^{6})$$

where *l_i_* corresponds to the bond lengths between a V ion and its nearest neighboring oxygen ion *i*, and *l*_min_ is the shortest V─O bond length within the coordination environment. A higher ECN generally reflects a more complete and less distorted local coordination geometry. As shown in Figure 8e, the GZVO model exhibits a higher ECN than pristine ZVO, indicating that the NIR‐associated interface helps maintain the V─O coordination environment closer to an ideal octahedral configuration. This reduced local distortion supports more stable Zn^2+^ insertion/extraction and helps suppress irreversible structural degradation.

The centrosymmetry parameter (CSP) was further calculated to evaluate 3D structural distortion around the V‐centered oxygen coordination environment (Figure 8f–h). The average CSP value decreases from 9.22 for pristine ZVO to 7.80 for the GZVO model, revealing lower local symmetry distortion after NIR integration. Together with the ECN result, the reduced CSP value indicates that the N‐rich interfacial component contributes to stabilizing the local VO_6_ coordination geometry. As shown in Figure S25, Bader charge analysis was performed to evaluate interfacial charge redistribution between the N‐rich component and the ZVO surface. The Bader charges of the surface Zn and V atoms were quantified for both GZVO and pristine ZVO, revealing increased Bader charge values for both Zn and V in GZVO compared with those in pristine ZVO. These variations demonstrate that integration with the N‐rich component modifies the electronic environment of the ZVO surface rather than producing simple unidirectional charge donation from GCN to ZVO. The resulting interfacial electronic interaction is expected to regulate surface charge redistribution during Zn^2+^ insertion and extraction and mitigate the associated local structural variations. Importantly, the Bader results are interpreted as evidence of electronic coupling at the GCN/ZVO interface and not as standalone proof of a permanent covalent Zn─N bond. Together with the N 1s binding‐energy shifts and electrochemical‐state‐dependent spectral evolution, these calculations support a dynamic Zn^2+^‐responsive interaction involving the polar nitrogen environments of GCN. Overall, the DFT results reveal the dual role of the NIR‐associated interface in regulating Zn^2+^ storage behavior. The calculated surface Zn‐removal energy of GZVO is −3.4 eV, which is ∼1.0 eV more favorable than that of pristine ZVO (−2.4 eV). This indicates that Zn species are more readily released from the NIR‐associated ZVO surface during the charge process. Experimentally, this energetic advantage agrees with the reduced electrochemical polarization of GZVO‐7 wt.%, as reflected by the smaller charge/discharge voltage hysteresis and lower interfacial resistance compared with pristine ZVO. Specifically, the GZVO‐7 wt.% electrode delivers a high reversible capacity of 258.93 mAh g^−1^ even at 10 A g^−1^ and recovers to 520.34 mAh g^−1^ when the current density returns to 0.5 A g^−1^, confirming rapid and reversible charge‐storage kinetics under high‐current operation. In addition to facilitating Zn release, the NIR‐associated interface also improves the local structural stability of the ZVO framework. The more negative average ICOHP value indicates stronger V─O bonding, while the increased ECN and reduced CSP reflect a more stable and less distorted local coordination environment. These theoretical descriptors quantitatively support the experimentally observed cycling durability: GZVO‐7 wt.% maintains 218.24 mAh g^−1^ after 3000 cycles at 5 A g^−^^1^, which is higher than ZVO (148.42 mAh g^−1^), GZVO‐5 wt.% (171.41 mAh g^−1^), and GZVO‐10 wt.% (70.07 mAh g^−1^) under identical conditions. Therefore, the ∼1.0 eV more favorable Zn‐removal energy explains the improved reversibility of Zn^2+^ extraction, whereas the strengthened V─O bonding and stabilized coordination environment account for the superior long‐term framework durability of the optimized GZVO‐7 wt.% cathode. More extensive calculations with models incorporating larger GCN fragments, periodic polymeric sheets, or multilayer structures could provide further insights into spatially resolved interfacial charge transfer and Zn^2^^+^ migration pathways. These extended models would be particularly valuable for elucidating the detailed surface ion‐exchange processes involving Zn^2^^+^ adsorption, migration, insertion, and extraction.

## 3 Conclusion

In summary, NIR‐integrated spinel ZVO cathodes were successfully constructed through solvothermal synthesis, Ar‐assisted calcination, and ultrasonication‐assisted physical integration with a GCN‐derived NIR. The optimized GZVO‐7 wt.% cathode retains detectable ZVO‐derived crystalline domains while establishing a regulated N‐rich interfacial architecture, leading to the best electrochemical performance among the investigated electrodes. GZVO‐7 wt.% delivers a high reversible capacity of 405.16 mAh g^−1^ after 150 cycles at 0.5 A g^−1^, excellent rate capability with 258.93 mAh g^−1^ retained at 10 A g^−1^, and stable long‐term cycling with 218.24 mAh g^−1^ after 3000 cycles at 5 A g^−1^, accompanied by nearly 100% Coulombic efficiency. The comparatively lower performance of GZVO‐5 wt.% and GZVO‐10 wt.% confirms that an optimized NIR content is essential for balancing interfacial regulation, Zn^2+^ accessibility, and active‐site utilization. The enhanced Zn^2+^ storage behavior of GZVO‐7 wt.% originates from the synergistic improvement of interfacial charge transfer, surface‐dominated charge‐storage kinetics, and stabilization of the local V─O coordination environment. CV kinetic analysis, in situ EIS–DRT, and GITT measurements demonstrate enhanced pseudocapacitive contribution, reduced polarization, and reversible Zn^2+^ storage behavior. Ex situ XRD/XPS analyses reveal a reversible Zn^2+^ insertion/extraction mechanism involving persistence and partial recovery of ZVO‐derived crystalline domains, surface V^5+^/V^4+^ redox evolution, local oxygen‐environment modulation, reversible N‐site‐mediated Zn^2+^ interfacial regulation, and dynamic cathode/electrolyte interphase evolution. DFT calculations further support the atomic‐level role of the NIR‐associated interface by revealing more favorable surface Zn‐removal energetics, strengthened V─O bonding, and reduced local coordination distortion. Overall, this work demonstrates that NIR‐guided interfacial engineering is an effective strategy for developing durable, high‐rate vanadate cathodes for AZIBs.

## 4 Experimental Section

### 4.1 Materials

Ammonium metavanadate (NH_4_VO_3_, ACS reagent, ≥ 99.0%), zinc nitrate hexahydrate (Zn(NO_3_)_2_·6H_2_O, reagent grade, 98%), urea (CO(NH_2_)_2_, BioXtra, pH 7.5–9.5), ethylene glycol (EG, C_2_H_6_O_2_, reagent grade, ≥ 99.5%), n‐methyl‐2‐pyrrolidone (NMP, 99.5%), and zinc trifluoromethanesulfonate (Zn(CF_3_SO_3_)_2_, 98%) were received from Sigma–Aldrich (South Korea). Polyvinylidene fluoride (PVDF, (CH_2_CF_2_)_n_) purchased from Daikin (South Korea). Zinc foil (0.1 mm thickness) and CR2032 coin cell components were sourced from MTI Korea (South Korea).

### 4.2 Synthesis of Spinel Zinc Vanadate (ZVO)

Ammonium metavanadate (0.116 g) was dissolved in a mixed solvent of deionized (DI) water (40 mL) and ethylene glycol (EG, 40 mL) at 80°C for 0.5 h under magnetic stirring. Subsequently, zinc nitrate hexahydrate (0.297 g) was added to the solution, and the mixture was continuously stirred until a golden‐yellow solution was obtained. These precursor quantities correspond to approximately 0.992 mmol of V and 0.998 mmol of Zn, giving a nominal Zn:V feeding ratio of approximately 1.01:1. This precursor ratio is Zn‐rich relative to the theoretical Zn:V ratio of 0.50:1 for stoichiometric ZnV_2_O_4_. Therefore, the precursor feeding ratio should not be interpreted as the final stoichiometry of the isolated product. The resulting solution was transferred into a 100 mL stainless‐steel autoclave and subjected to solvothermal treatment at 200°C for 30 h. After naturally cooling to room temperature, the precipitate was collected and washed alternately with DI water and ethanol three times by centrifugation. The obtained product was dried at 80°C for 12 h. The dried precursor was then calcined at 800°C for 2 h under continuous Ar flow in a tube furnace at a heating rate of 5°C min^−1^ to obtain a Zn‐rich zinc vanadate material with a dominant cubic spinel ZnV_2_O_4_ crystalline phase, hereafter denoted as ZVO.

### 4.3 Preparation of Nitrogen‐Rich Interfacial Regulator‐Integrated Zinc Vanadate (GZVO) Composites

Graphitic carbon nitride (g‑C_3_N_4_, GCN) was synthesized by thermal polymerization of urea. Briefly, 5 g of urea was placed in a covered alumina crucible and calcined at 550°C for 4 h under continuous N_2_ flow at a heating rate of 5°C min^−1^. After naturally cooling to room temperature, the resulting pale‐yellow product was collected and ground into a fine powder using an agate mortar and pestle. The obtained GCN powder was stored in a desiccator before use.

The GCN‐derived nitrogen‐rich interfacial regulator (NIR)‐integrated ZVO composites were prepared through an ultrasonication‐assisted physical integration strategy. First, a predetermined amount of GCN powder was dispersed in 10 mL of DI water and ultrasonicated for 2 h to obtain a uniform suspension. A calculated amount of as‐synthesized ZVO powder was then added to the GCN suspension and magnetically stirred for 1 h at room temperature to promote intimate interfacial association between ZVO nanoparticles and the NIR backbone. The resulting mixture was dried at 80°C overnight in an air oven. Because the complete suspension was directly dried and no centrifugation, filtration, washing, or other solid‐separation procedure was performed after combining GCN and ZVO, no deliberate selective removal of either solid component occurred during composite preparation. The dried solid was collected and gently ground into a fine powder for use as the composite cathode material.

Three composites with different GCN contents were prepared by varying the GCN weight fraction while maintaining a fixed total solid mass of 500 mg. The composites containing 5, 7, and 10 wt.% GCN were designated as GZVO‐5 wt.% (25 mg GCN and 475 mg ZVO), GZVO‐7 wt.% (35 mg GCN and 465 mg ZVO), and GZVO‐10 wt.% (50 mg GCN and 450 mg ZVO), respectively. Accordingly, the GCN contents represent gravimetrically controlled nominal loadings. Based on the ideal nitrogen mass fraction of g‐C_3_N_4_ (60.86 wt.%), the corresponding calculated nominal nitrogen contents of GZVO‐5 wt.%, GZVO‐7 wt.%, and GZVO‐10 wt.% are 3.04, 4.26, and 6.09 wt.%, respectively. These values increase proportionally with the weighed GCN content and are reported as calculated nominal nitrogen contents rather than experimentally measured bulk nitrogen concentrations. For comparison, pristine ZVO without GCN integration was prepared and evaluated under identical conditions. Among these electrodes, GZVO‐7 wt.% exhibited the most balanced electrochemical performance and was selected as the representative sample for detailed structural and mechanistic investigation. Owing to its nitrogen‐rich layered framework, the GCN‐derived NIR is expected to provide polar coordination sites and interfacial contact pathways, thereby regulating the local ionic/electronic environment without producing a detectable change in the dominant ZVO crystalline phase within the detection limit of PXRD.

### 4.4 Physicochemical Characterization

The morphology and microstructure of the synthesized materials were examined using scanning electron microscopy (SEM, SU8600, Hitachi) equipped with energy‐dispersive x‐ray spectroscopy (EDS) and transmission electron microscopy (TEM, JXA‐8530F, JEOL). The crystalline phase and structural composition were analyzed by x‐ray diffraction (XRD, EMPYREAN, PANalytical) using Cu Kα radiation (λ = 0.15406 nm) over a 2θ range of 10°–80°. Functional groups were identified by Fourier‐transform infrared spectroscopy (FT‐IR, Frontier, PerkinElmer) in the wavenumber range of 400–4000 cm^−1^. The surface chemical states of the constituent elements and defect‐related oxygen species were investigated by x‐ray photoelectron spectroscopy (XPS, NEXSA, Thermo Fisher Scientific) using Al Kα radiation.

### 4.5 Cell Fabrication

Cathode electrodes were fabricated by mixing the active material, Super P carbon black, and PVDF binder at a mass ratio of 7:2:1. The mixed powders were dispersed in NMP and homogenized using a planetary centrifugal mixer (Thinky ARE‐310) to form a uniform slurry. The slurry was coated onto carbon cloth substrates and dried at 80°C overnight under vacuum to obtain cathode electrodes with an active material mass loading of 1.3–1.5 mg cm^−2^. CR2032 coin cells were assembled in ambient air using the prepared cathode, Zn foil anode, glass fiber separator (GF/D, Whatman), and 100 µL of aqueous electrolyte. The electrolyte was prepared by dissolving 3 M zinc trifluoromethanesulfonate (Zn(CF_3_SO_3_)_2_) in DI water.

### 4.6 Electrochemical Measurement

All electrochemical measurements were performed in a temperature‐controlled battery chamber. Galvanostatic charge–discharge (GCD) tests and cycling performance evaluations were conducted using a battery testing system (Neware, MIHW‐200‐160CH B) within a voltage window of 0.2–1.8 V vs Zn/Zn^2+^. Cyclic voltammetry (CV) measurements were carried out at different scan rates to evaluate electrochemical kinetics and capacitive contribution. Electrochemical impedance spectroscopy (EIS) was performed using a Bio‐Logic VSP potentiostat over a frequency range of 100 kHz to 0.01 Hz with an AC amplitude of 10 mV. In situ EIS was conducted during the initial discharge/charge process at selected potential steps to monitor the evolution of interfacial resistance. Distribution of relaxation times (DRT) analysis was performed using MATLAB to deconvolute frequency‐dependent resistance components and identify individual polarization contributions.

The galvanostatic intermittent titration technique (GITT) was employed to determine the apparent Zn^2+^ diffusion coefficient (D_Zn_). A current pulse of 0.1 A g^−1^ was applied for 10 min, followed by a 1 h relaxation interval within the voltage range of 0.2–1.8 V. D_Zn_ was calculated using the following equation:

$$\;D_{Zn}=\frac{4}{\pi \tau }\;{\left(\frac{m_{B}V_{M}}{M_{B}S}\right)}^{2}{\left(\frac{\Delta E_{s}}{\Delta E_{\tau }}\right)}^{2}$$

where τ is the current‐pulse duration (s), m_B_ is the mass of active material (g), V_M_ is the molar volume of the electrode material (cm^3^ mol^−1^), M_B_ is the molar mass (g mol^−1^), S is the geometric area of the electrode (cm^2^), ΔE_s_ is the steady‐state potential change induced by the current pulse, and ΔE_τ_ is the transient potential change during the current pulse after iR‐drop correction.

### 4.7 Computational Details

Density functional theory (DFT) calculations were performed using the Vienna Ab initio Simulation Package (VASP) [53]. The exchange–correlation interactions were described using the Perdew–Burke–Ernzerhof generalized gradient approximation (GGA‐PBE). To account for the localized V 3d electrons, the GGA+U method introduced by Dudarev et al. [54] was employed, with an effective U value of 3.25 eV for V. Projector‐augmented‐wave (PAW) potentials were used to describe the ion–electron interactions, with valence configurations of Zn 3d^10^4s^2^, V 3s^2^3p^6^3d^4^4s^1^, O 2s^2^2p^4^, C 2s^2^2p^2^, and N 2s^2^2p^3^. The plane‐wave cutoff energy was set to 500 eV based on convergence testing. The convergence criterion for electronic self‐consistent iterations was set to 10^−6^ eV, and all structures were fully relaxed until the residual forces on each atom were less than 0.01 eV Å^−1^. Considering the weak interfacial interactions between ZVO and GCN, van der Waals correction was included during structural relaxation and total‐energy calculations. A vacuum layer of at least 15 Å was introduced perpendicular to the slab surface to avoid artificial interactions between periodic images.

## Author Contributions

**Ashok Kumar Kakarla**: conceptualization, methodology, investigation, validation, data curation, writing – original draft, writing – review and editing. **Incheol Jeong**: writing – original draft, writing – review and editing, data curation, methodology, validation, investigation. **Chanho Lee**: validation, formal analysis, investigation. **SeungEun Yu**: validation, formal analysis. **Ryeowon Kang**: validation, formal analysis, investigation. **Binna Kim**: investigation, validation, formal analysis. **Wooseop Jo**: investigation, validation, formal analysis. **San Moon**: methodology, software, formal analysis. **Patrick Joohyun Kim**: methodology, software, formal analysis. **Seho Sun**: methodology, software, formal analysis. **Chanho Kim**: methodology, software, formal analysis. **Ki‐Min Roh**: project administration, resources, writing – original draft, writing – review and editing, funding acquisition, conceptualization. **Junghyun Choi**: project administration, resources, writing – original draft, writing – review and editing, funding acquisition. **Dongsoo Lee**: project administration, resources, writing – original draft, writing – review and editing, funding acquisition, conceptualization, supervision, visualization.

## Conflicts of Interest

The authors declare no conflicts of interest

## Supporting information




**Supporting File**: advs78612‐sup‐0001‐SuppMat.docx.

## Data Availability

The data that support the findings of this study are available from the corresponding author upon reasonable request.
