# Supplementary material for: Coupling Fast Zn2+ Kinetics With V–O Framework Stabilization via Nitrogen‐Rich Interfacial Engineering of Spinel ZnV2O4
Source: Adv Sci (Weinh). 2026 Nov 9:e78612. Online ahead of print. doi: 10.1002/advs.78612 (PMC13749001; doi:10.1002/advs.78612)
Supplement: Supplementary file 1 [file ADVS-9999-e78612-s001.docx]

**Supporting Information**

**Coupling Fast Zn^2+^ Kinetics with V–O Framework Stabilization via Nitrogen-Rich Interfacial Engineering of Spinel ZnV_2_O_4_**

*Ashok Kumar Kakarla^a,^*^†^*, Incheol Jeong^b,^*^†^*,Chanho Lee^a^, SeungEun Yu^a^, Ryeowon Kang^a^, Binna Kim^a^, Wooseop Jo^a^, San Moon^c^, Patrick Joohyun Kim^d^, Seho Sun^e^, Chanho Kim^f^, Ki-Min Roh^b,^*, Junghyun Choi^a,^*, Dongsoo Lee^a,^**

^a^ School of Chemical, Biological & Battery Engineering, Gachon University, Gyeonggi-do 13120, Republic of Korea

E-mail: [junghchoi@gachon.ac.kr](mailto:junghchoi@gachon.ac.kr), [dslee9117@gachon.ac.kr](mailto:dslee9117@gachon.ac.kr)

^b^ Resources Utilization Research Center, KIGAM, Daejeon 34132, Republic of Korea

E-mail: [kmroh@kigam.re.kr](mailto:kmroh@kigam.re.kr)

^c^ Advanced Battery Research Center, Korea Research Institute of Chemical Technology, Daejeon, Republic of Korea

^d^ Department of Chemical and Biological Engineering, Sookmyung Women’s University100 Cheongpa-ro 47-gil, Yongsan-gu, Seoul 04310, Republic of Korea^e^ School of Chemical Engineering, Yeungnam University, Gyeongsan, Republic of Korea

^f^ Chemical Science Division, Oak Ridge National Laboratory, Oak Ridge, Tennessee, USA

**Figure S1.** (a) Dilution-corrected ICP-OES concentrations of V and Zn in the submitted ZVO and GZVO sample solutions and (b) calculated Zn molar ratios compared with the theoretical value of 0.50 for stoichiometric ZnV_2_O_4_.

**Figure S2.** Survey scan spectrum of ZVO and GZVO composite materials.

**Figure S3.** High-resolution Zn 2p XPS spectra of (a) GZVO-5 wt% and (b) GZVO-10 wt%.

**Figure S4.** High-resolution V 2p/O 1s XPS spectra of (a) GZVO-5 wt% and (b) GZVO-10 wt%.

**Figure S5.** High-resolution N 1s XPS spectra of (a) ZVO, (b) GZVO-5 wt%, and (c) GZVO-10 wt%.

**Figure S6.** N 1s XPS spectrum of GCN sample

**Figure S7.** High-resolution C 1s XPS spectra of (a) GZVO-5 wt% and (b) GZVO-10 wt%.

**Figure S8.** N_2_ adsorption–desorption isotherms of ZVO, GZVO-5 wt%, GZVO-7 wt%, and GZVO-10 wt% samples.

**Figure S9.** Initial five CV curves of ZVO, GZVO-5 wt%, GZVO-7 wt%, and GZVO-10 wt% cathodes at 0.5 mV s^−1^, showing the electrochemical activation behavior and redox reversibility during Zn^2+^ insertion/extraction.

**Figure S10.** Initial five galvanostatic charge/discharge profiles of ZVO, GZVO-5 wt%, GZVO-7 wt%, and GZVO-10 wt% cathodes at 0.5 A g^−1^, showing the activation behavior and voltage-profile stability during early Zn^2+^ insertion/extraction cycles.

**Figure S11.** Differential capacity (dQ/dV) profiles of ZVO, GZVO-5 wt%, GZVO-7 wt%, and GZVO-10 wt% cathodes during the initial five cycles at 0.5 A g^−1^, showing the redox evolution and reaction reversibility during Zn^2+^ insertion/extraction.

**Figure S12.** Cycling performance of GZVO-7 wt% cathodes with active-material mass loadings of approximately 1.5, 5, and 10 mg cm^−2^ at 0.5 A g^−1^ for 100 cycles.

**Figure S13.** Post-cycling structural and morphological characterization of the GZVO-7 wt% cathode. (a) XRD patterns after 1000 and 3000 cycles, confirming retention of the ZVO phase. (b–e) FE-SEM images after 1000 and 3000 cycles at different magnifications. (f–j) EDS elemental mapping images after cycling, showing the distribution of Zn, V, O, N, and C.

**Figure S14.** (a–c) TEM images of the GZVO-7 wt% electrode recovered after 3000 cycles at 5 A g^−1^ at different magnifications. (d) High-resolution TEM image and (e) SAED pattern. (f–j) STEM–EDS elemental maps of V, Zn, C, N, and O, respectively, showing that the N-containing component remains spatially associated with the Zn/V/O-rich particle regions after long-term cycling.

**Figure S15.** Zn^2+^ storage kinetics of (a) CV plots at different scan rates, (b) corresponding (b)-value fitting plots of redox peaks, and (c) capacitive and diffusion-controlled contribution ratios at various scan rates for the GZVO-5 wt% electrode.

**Figure S16.** Zn^2+^ storage kinetics of (a) CV plots at different scan rates, (b) corresponding (b)-value fitting plots of redox peaks, and (c) capacitive and diffusion-controlled contribution ratios at various scan rates of the GZVO-10 wt% electrode.

**Figure S17.** (a) GITT potential profiles and (b) corresponding apparent Zn^2+^ diffusion coefficients calculated from the GITT curves of the GZVO-5 wt% electrode.

**Figure S18.** (a) GITT potential profiles and (b) corresponding apparent Zn^2+^ diffusion coefficients calculated from the GITT curves of the GZVO-10 wt% electrode.

**Figure S19.** In situ EIS and DRT analysis of ZVO. (a, b) EIS spectra during discharge and charge. (c) Corresponding DRT profiles. (d) 2D DRT contour map.

**Figure S20.** In situ EIS and DRT analysis of GZVO-5 wt%. (a, b) EIS spectra during discharge and charge. (c) Corresponding DRT profiles. (d) 2D DRT contour map.

**Figure S21.** In situ EIS and DRT analysis of GZVO-10 wt%. (a, b) EIS spectra during discharge and charge. (c) Corresponding DRT profiles. (d) 2D DRT contour map.

**Figure S22.** XPS survey spectra of GZVO-7 wt% electrodes in the pristine, fully discharged at 0.2 V, and fully charged at 1.8 V states, confirming the presence of C, N, V, O, and Zn during Zn^2+^ insertion/extraction.

**Figure S23.** High-resolution N 1s XPS spectra of GZVO-7 wt% electrodes in the pristine, fully discharged at 0.2 V, and fully charged at 1.8 V states, showing the evolution of C–N=C, N–(C)_3_/Zn^2+^···N-interacting sites, and –NH components during Zn^2+^ insertion/extraction.

**Figure S24.** High-resolution C 1s XPS spectra of GZVO-7 wt% electrodes in the pristine, fully discharged at 0.2 V, and fully charged at 1.8 V states, showing the evolution of C–C, C–N, N–C=N, and electrolyte-derived CF_3_/CF_x_-containing components during cycling.

**Figure S25.** Bader charge analysis of Zn and V on ZVO and GZVO.

**Table S1.** Measured ICP-OES Zn/V ratios and gravimetrically calculated nominal GCN and nitrogen contents.

| **Sample** | **Measured Zn:V molar ratio** | **Nominal GCN loading** | **Calculated nominal N content** |
| --- | --- | --- | --- |
| **ZVO** | 1.260:1 | 0 wt% | 0 wt% |
| **GZVO-5 wt%** | 1.251:1 | 5 wt% | 3.04 wt% |
| **GZVO-7 wt%** | 1.527:1 | 7 wt% | 4.26 wt% |
| **GZVO-10 wt%** | 1.266:1 | 10 wt% | 6.09 wt% |

**Table S2.** Fitted component ratios of O 1s, N 1s, and C 1s XPS spectra for GZVO-7 wt% at the pristine, fully discharged, and fully charged states.

| **Core level** | **Component assignment** | **Pristine (%)** | **Discharge to 0.2 V (%)** | **Charge to 1.8 V (%)** |
| --- | --- | --- | --- | --- |
| O 1s | Lattice oxygen | 45.22 | 23.43 | 21.60 |
| O 1s | Defect-related oxygen | 42.19 | 39.01 | 52.06 |
| O 1s | Adsorbed oxygen | 12.59 | 37.56 | 26.34 |
| N 1s | C-N=C | 32.10 | 16.48 | 74.88 |
| N 1s | Tertiary N–(C)_3_ | 47.61 | 58.98 | 6.04 |
| N 1s | Terminal/protonated N species | 20.29 | 24.54 | 19.08 |
| C 1s | C–C/C=C | 37.12 | — | 37.61 |
| C 1s | C–N | 44.10 | 22.38 | 45.62 |
| C 1s | N–C=N | — | 14.22 | — |
| C 1s | CF_3_-containing species | — | 7.85 | — |
| C 1s | CF_x_-containing species | — | 5.97 | — |
| C 1s | Residual carbon species | 18.78 | 49.58 | 16.77 |
